# The Effect of Decision Fatigue on Job Stress in Midwives: The Mediating Role of Psychological Capital and the Moderating Effect of Perceived Organizational Support

**DOI:** 10.1155/jonm/4433124

**Published:** 2026-05-29

**Authors:** Xianying Lu, Yimin Zhang, Zhirong Ren, Xinyu Chen, Dingxi Bai, Jing Gao, Chaoming Hou

**Affiliations:** ^1^ School of Nursing, Chengdu University of Traditional Chinese Medicine, Chengdu, China, cdutcm.edu.cn; ^2^ The Fifth Affiliated People’s Hospital, Chengdu University of Traditional Chinese Medicine, Chengdu, China, cdutcm.edu.cn

**Keywords:** decision fatigue, job stress, midwives, perceived organizational support, psychological capital

## Abstract

**Background:**

Midwives face escalating decision fatigue (DF) and job stress. If left unaddressed, these issues may impair their mental health and clinical performance, compromise maternal and neonatal safety, and exacerbate workforce shortages, making it urgent to explore the underlying influencing mechanisms. This study explored the status of job stress among midwives, analyzed the correlations between DF, psychological capital (PsyCap), perceived organizational support (POS), and job stress and further examined the mediating role of PsyCap as well as the moderating role of POS on the relationship between DF and job stress.

**Methods:**

A multicenter cross‐sectional survey was conducted among midwives from 146 Baby‐Friendly Hospitals in Sichuan, China, between October 2024 and September 2025. Using multistage stratified cluster sampling, 1263 midwives completed questionnaires on DF, PsyCap, POS, and job stress. SPSS 26.0 was applied for descriptive statistics, one‐way ANOVA, and Pearson correlation analysis, while Mplus 8.3 was used to conduct mediation and moderation analyses.

**Results:**

The total scores (mean ± SD) of job stress, DF, PsyCap, and POS among midwives were 90.45 ± 19.57, 20.58 ± 5.92, 88.83 ± 14.08, and 43.71 ± 9.98, respectively. One‐way ANOVA showed significant differences in job stress scores across age, ethnicity, hospital ownership, hospital level, employment type, work experience, and monthly night shifts (all *p* < 0.05). Pearson correlation analysis revealed that job stress was positively correlated with DF (*r* = 0.648, *p* < 0.01) and negatively correlated with PsyCap (*r* = −0.673, *p* < 0.01) as well as POS (*r* = −0.342, *p* < 0.01). PsyCap partially mediated the relationship between DF and job stress (indirect effect = 0.258, 95% CI [0.223, 0.300]). Moreover, POS negatively moderated the path between PsyCap and job stress (*γ* = −0.165, *p* < 0.001), indicating a significant moderated mediation effect.

**Conclusion:**

DF directly increases job stress among midwives and also exerts an indirect effect through PsyCap, with this mediation being moderated by POS. Targeted interventions to reduce DF, enhance PsyCap, and strengthen POS may alleviate job stress and improve midwives’ well‐being.

**Implication for Nursing Management:**

Nursing managers should formulate targeted measures to reduce midwives’ DF, improve their PsyCap, and strengthen organizational support. Such comprehensive strategies may effectively relieve job stress, promote occupational well‐being, and stabilize the midwifery workforce in Baby‐Friendly Hospitals.

**Summary**
• What is already known
◦ Decision fatigue is an identified stressor for healthcare professionals.
◦ Psychological capital and perceived organizational support are known to mitigate job stress.
• What this paper adds
◦ Evidence that decision fatigue is directly linked to higher job stress among midwives in China.
◦ Identification of psychological capital as a key mediator in the relationship between decision fatigue and job stress.
◦ Demonstration that perceived organizational support moderates this mediating pathway, buffering the impact on stress.

## 1. Introduction

Midwives are pivotal to global maternal and neonatal health outcomes, capable of providing up to 87% of essential care when adequately educated and supported [1]. However, this profession faces escalating work pressure due to demographic shifts toward aging populations and declining fertility rates [2], leading to policy changes aimed at encouraging births. These shifts have resulted in an increased proportion of high‐risk pregnancies (e.g., advanced maternal age and multiparity) and heightened demands for prenatal, obstetric, and pediatric care [3, 4]. However, midwives worldwide face escalating job stress, driven by factors such as increasing service demands, complex clinical scenarios, and the high stakes of obstetric decision‐making [5]. This stress not only compromises midwives’ well‐being but also undermines the quality of care, creating a critical challenge for healthcare systems globally [6]. Understanding the mechanisms driving this stress is therefore essential for developing effective interventions.

Within this context, decision fatigue (DF) emerges as a significant and specific source of stress for midwives. The autonomous and time‐sensitive nature of midwifery practice necessitates frequent, rapid, and often complex clinical decisions during labor and delivery [7]. DF refers to the deterioration in decision‐making quality and self‐regulatory capacity resulting from the cumulative cognitive burden of repeated decision‐making [8]. It impairs cognitive functions such as attention, memory, judgment, and reasoning [9] and is linked to reduced self‐control [9, 10]. Previous studies have shown that DF can affect the cognitive ability of medical staff, reduce the quality of final medical decisions [10], potentially compromising patient safety [10, 11], and birth outcomes [12]. While strategies such as strategic breaks or task delegation are suggested to mitigate DF [11, 13, 14], their feasibility is often limited in the continuous, high‐demand context of clinical midwifery.

**FIGURE 1:**
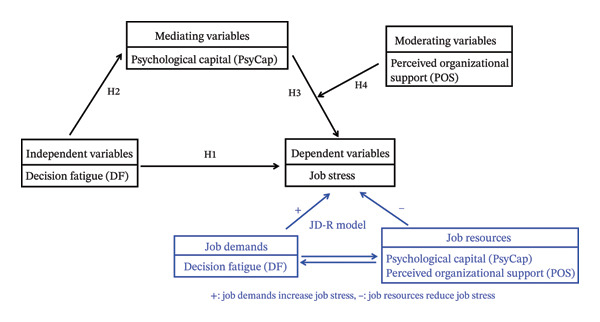
Research structure.

Against this backdrop, the Job Demands–Resources (JD‐R) model provides a robust theoretical framework to explore the mechanisms underlying midwives’ job stress [15] (Figure 1). This model posits that workplace factors can be categorized into job demands (physically or psychologically taxing elements that deplete resources) and job resources (factors that buffer stress and enhance coping capacity). Two core processes drive outcomes: a “health impairment process,” where excessive job demands lead to burnout and stress [16, 17], and a “motivational process,” where job resources foster engagement and resilience [18]. Applied to midwifery, DF aligns with job demands, as repeated high‐stakes decisions deplete cognitive resources and contribute to stress. In contrast, psychological capital (PsyCap) and perceived organizational support (POS) qualify as critical job resources, with the potential to mitigate the adverse effects of such demands [19].

PsyCap, a construct encompassing hope, optimism, resilience, and self‐efficacy, emerges as a key intrinsic resource in this context [20]. It enhances coping abilities, reduces burnout, and buffers against stress by fostering a proactive mindset [21]. Evidence suggests that DF can deplete these resources, negatively impacting PsyCap levels [22, 23]. Conversely, PsyCap is a well‐established protective factor against job stress; higher PsyCap enables individuals to navigate stress more effectively [24, 25]. This suggests that PsyCap may act as a crucial mediating mechanism: DF depletes PsyCap, and this depletion subsequently exacerbates the experience of job stress. Investigating this mediating pathway offers a novel perspective on how DF translates into heightened stress for midwives. Additionally, POS, the employees’ perception of organizational care, recognition, and responsiveness to their needs [26], functions as an extrinsic job resource. High POS fosters greater work engagement and buffers against fatigue and stress [27]. Crucially, POS is positively associated with PsyCap, suggesting it may strengthen the beneficial effects of PsyCap [28, 29]. Within the JD‐R framework, POS represents a key contextual factor that may moderate the interplay between demands (DF), resources (PsyCap), and outcomes (job stress), shaping the effectiveness of resource‐based coping.

Unaddressed DF and chronic job stress among midwives not only erode individual mental well‐being and professional competence but also lead to careless clinical judgment, reduced obstetric care quality, and elevated risks of adverse maternal and neonatal outcomes. Although midwives’ job stress has been studied, the existing research often focuses on individual or limited sets of factors. A significant gap remains in understanding the integrated mechanism linking DF, PsyCap, POS, and job stress. Specifically, the potential mediating role of PsyCap in the DF–job stress relationship, and the potential moderating role of POS in the PsyCap–job stress link and/or the overall mediated pathway, remain unexplored in the context of midwifery. Therefore, grounded in the JD‐R model and leveraging insights from positive psychology, this study investigates a moderated mediation model (Figure 1). We propose the following.

 H1: DF has a positive direct effect on job stress (consistent with the health impairment process);
 H2: DF has a negative effect on PsyCap;
 H3: PsyCap has a negative effect on job stress;
 H4: PsyCap mediates the association between DF and job stress;
 H5: POS moderates the mediating effect, such that higher POS strengthens PsyCap’s ability to reduce stress.

By elucidating these interconnected mechanisms, this study aims to provide a more comprehensive theoretical understanding of the formation mechanisms of midwives’ job stress. In addition, the findings offer evidence‐based references for nurse leaders to formulate rational management and intervention strategies that target the alleviation of DF, the improvement of PsyCap, and the reinforcement of POS. Ultimately, these insights inform the development of targeted measures to relieve job stress, promote midwives’ long‐term well‐being and workforce stability, and sustain the delivery of high‐quality maternal and neonatal care.

## 2. Methods and Materials

### 2.1. Study Design

A multicenter, cross‐sectional study using structural equation modeling (SEM) was adopted to explore the relationships among DF, PsyCap, POS, and job stress in midwives. The study was conducted in accordance with the Strengthening the Reporting of Observational Studies in Epidemiology (STROBE) guidelines. Ethical approval was obtained from the Ethics Committee of the Fifth People’s Hospital of Chengdu (No: 2024‐058‐01).

### 2.2. Participants and Setting

Participants were recruited from October 2024 to September 2025 across 146 Baby‐Friendly Hospitals (designated by the National Health Commission of China) covering 16 prefecture‐level cities in Sichuan Province, China. A multistage stratified cluster sampling approach was used: (1) Stage 1 (city stratification): Sichuan Province was divided into five economic zones, and finally 16 cities/autonomous prefectures were selected proportional to regional GDP (Chengdu Plain Economic Zone: 8; Southern Sichuan Economic Zone: 3; Northeast Sichuan Economic Zone: 3; Panxi: 1; West Sichuan Economic Zone: 1) by random sampling. (2) Stage 2 (hospital stratification): Hospitals were sampled by tier (Primary: Secondary: Tertiary = 2:5:3) and accreditation level (Class A/B), and finally 22 primary hospitals (20 Class A, 2 Class B), 79 secondary hospitals (46 Class A, 33 Class B), 45 tertiary hospitals (26 Class A, 19 Class B) were selected. (3) Third stage (midwife selection): All eligible midwives from the 146 selected hospitals were invited to participate. The detailed sampling strategies are shown in Appendix 1.

#### 2.2.1. Inclusion and Exclusion Criteria

Inclusion criteria include the following: (1) hold a valid nurse practicing certificate and a maternal and child health technical qualification certificate, or hold a nurse practicing certificate and be currently engaged in midwifery work and (2) provide informed consent and voluntarily participate in the study. Exclusion criteria include the following: (1) interns, advanced trainees, or standardized training personnel; (2) midwives absent from work during the survey period due to study leave, sick leave, maternity leave, or other reasons; (3) midwives with a self‐reported history of psychiatric disorders (past or present); (4) midwives with chronic medical conditions (e.g., hypertension, diabetes, and thyroid disorders).

#### 2.2.2. Sample Size

The sample size was determined based on the number of items in the study scales, with a recommended ratio of 10–20 participants per item for multivariate analysis [30]. The study included 77 items across four scales (35 for job stress, 9 for DF, 20 for PsyCap, and 13 for POS), yielding an estimated sample size of 770–1540. Considering potential invalid responses, a 20% expansion was applied, resulting in a target sample size of 924–1848.

### 2.3. Measures

All instruments demonstrated strong reliability in pretesting (Cronbach’s *α* > 0.80) and prior validation studies.

#### 2.3.1. General Information Questionnaire

Based on the factors influencing work stress among midwives, the theoretical framework of the job demands‐resources (JD‐R) model, and the local clinical context, the general information questionnaire was developed by the research team, which included the principal investigator, nursing managers, midwifery experts, statistician, and graduate nursing students. The final questionnaire comprised 15 items, including demographic characteristics (age, gender, ethnicity, educational level, marital status, and fertility status) and work‐related characteristics (hospital type, hospital nature, hospital grade, employment type, work experience, professional title, professional position, monthly night shifts, and monthly income). Content validity was reviewed by two senior clinical midwifery managers. Face validity and item clarity were assessed during a pilot study with 64 midwives, which led to the revision of two items (age and work years) to improve logical consistency. This questionnaire was used for descriptive purposes and to identify potential covariates.

#### 2.3.2. Chinese Nurse Job Stressors Scale

The job stress was measured by the Chinese Nurse Job Stressors Scale developed by Grey‐Toft & Anderson and Wheeler & Riding and translated into a Chinese version by Li and Liu [31]. This 35‐item scale includes 5 dimensions: nursing profession and career issues, workload and time pressure, resource and environmental problems, patient care and interaction, and interpersonal relationships and management issues. Items are rated on a 4‐point Likert scale (1 = *no stress*, 4 = *severe stress*), with total scores ranging from 35 to 140. Higher scores indicate greater job stress. Previous studies have confirmed that the scale has good internal consistency, with Cronbach’s *α* coefficients of 0.80–0.94 for the total scale and each dimension [32–34]. In this study, the internal consistency of Cronbach’s *α* coefficient was 0.965.

#### 2.3.3. Decisional Fatigue Scale (DFS)

The DF was measured by the DFS developed by Hickman et al. [35] and validated in Chinese healthcare professionals by Yang et al. [36]. The 9‐item unidimensional scale uses a 4‐point Likert scale (0 = *completely disagree*, 3 = *completely agree*), with total scores ranging from 0 to 27. Higher scores indicate higher decisional fatigue. Yang et al. reported that the Cronbach’s *α* coefficient of DFS was 0.933, which confirmed its good internal consistency [36]. The Cronbach’s *α* coefficient in this study was 0.936.

#### 2.3.4. Nurse Psychological Capital Questionnaire (PCQ‐R)

The PsyCap was measured by the Nurse PCQ‐R revised by Luo and Hao based on the Chinese version of PCQ‐R [21], adapted for nursing settings. This 20‐item scale includes 4 dimensions: self‐efficacy (Items 1–6), hope (Items 7–12), resilience (Items 13–17), and optimism (Items 18–20). Items are rated on a 6‐point Likert scale (1 = *strongly disagree*, 6 = *strongly agree*). Total scores are categorized as ≤ 2.25 (*very low*), 2.26–3.50 (*low*), 3.51–4.75 (*moderate*), and > 4.75 (*high*). Previous reliability test showed that the Cronbach’s *α* coefficients of the total scale and its four dimensions ranged from 0.718 to 0.923, indicating good internal consistency [37]. The Cronbach’s *α* coefficient in this study was 0.945.

#### 2.3.5. Nurse POS Scale

The POS as measured by the Nurse POS Scale modified by Zuo [38] from Chen’s POS scale [39], adapted for nursing. This 13‐item scale includes 2 dimensions: emotional support (Items 1–10) and instrumental support (Items 11–13). Items are rated on a 5‐point Likert scale (1 = *strongly inconsistent*, 5 = *strongly consistent*), with total scores ranging from 13 to 65. Higher scores indicate higher POS. The psychometric properties of this scale have been evaluated in Chinese nursing samples, with a reported Cronbach’s *α* of 0.906 for the total scale [40]. The Cronbach’s *α* coefficient in this study was 0.944.

### 2.4. Data Collection Method

#### 2.4.1. Pilot Study

Before the actual data were collected, a pretest of the data collection instrument was conducted to ensure the appropriateness of the questionnaire. A pilot survey was conducted in September 2024 with 64 midwives from 4 hospitals in Chengdu (2 tertiary Grade A general hospitals, 1 tertiary Grade B maternal and child hospital, and 1 secondary specialized hospital). All 64 responses were valid, and the Cronbach’s *α* coefficients of all scales exceeded 0.8, indicating good reliability. The average completion time was 10 min.

#### 2.4.2. Formal Data Collection

Electronic questionnaires (via Wenjuanxing, a professional online survey platform) were used, combined with online and on‐site data collection. Before distribution, researchers obtained approval from hospital department heads and explained the study’s purpose, procedures, and confidentiality policies. Participants accessed the questionnaire after confirming informed consent (via an online checkbox).

### 2.5. Statistical Analysis

Data were analyzed using SPSS 26.0 and Mplus 8.3. Descriptive statistics were presented as frequencies (percentages) for categorical variables, and as means ± standard deviations or medians (interquartile ranges) for continuous variables, based on their distribution. Normality was assessed using skewness and kurtosis statistics. Common method bias was evaluated through an exploratory factor analysis (EFA): First, the Kaiser–Meyer–Olkin (KMO) test and Bartlett’s test of sphericity were performed (a KMO value > 0.6 and significant Bartlett’s test, *p* < 0.05, indicating suitability for factor analysis), followed by Harman’s single‐factor test where all scale items were included in an EFA, with CMB deemed nonsevere if the variance explained by the first factor was < 40%. For group difference analyses, independent *t*‐tests or one‐way ANOVA was used for normally distributed data, and Mann–Whitney U or Kruskal–Wallis H tests was used for non‐normally distributed data. Relationships among the main study variables (DF, PsyCap, POS, and job stress) were assessed using Pearson correlation analysis. To test the five research hypotheses (including the predictive relationships and the proposed mediation and moderation effects), SEM and latent moderated structural equation model (LMS) were constructed in Mplus 8.3: SEM was used to test the mediating role of PsyCap, LMS was used to test the moderating role of POS, all variables were mean‐centered to reduce multicollinearity, interaction terms (PsyCap × POS) were created to examine moderation effects, and the bootstrap method (5000 resamples, 95% confidence interval) was used to assess the significance of mediation and moderation effects, with effects considered significant if the 95% CI did not include 0.

## 3. Results

### 3.1. Participant Characteristics

A total of 1366 questionnaires were distributed to midwives, with 1263 valid responses (effective response rate: 92.46%). Invalid responses (*n* = 103) were excluded due to uniform answers (*n* = 22), completion time < 3 min (*n* = 67), illogical entries (e.g., 21‐year‐old chief nurses, *n* = 5), or patterned responses (*n* = 9). The final sample comprised 1263 midwives.

Their demographic and occupational characteristics are detailed in Appendix 2. Briefly, most midwives were aged 31–40 years (46.2%, *n* = 583), female (97.8%, *n* = 1235), and Han Chinese (95.0%, *n* = 1200). The majority held a bachelor’s degree (78.2%, *n* = 988) and worked in general hospital (73.2%, *n* = 924) or public hospital (79.6%, *n* = 1005), primarily at the tertiary A level (38.6%, *n* = 487). Most were contract‐employed (78.0%, *n* = 985) with 5–10 years (34.5%, *n* = 436) or 11–20 years (34.8%, *n* = 439) of work experience and held the title of nurse‐in‐charge (47.0%, *n* = 594).

### 3.2. Scale Reliability

The internal consistency of the four scales used in this study was excellent. As shown in Table 1, Cronbach’s *α* coefficients for the total scales and all subscales exceeded 0.80, indicating strong reliability.

**TABLE 1:** Reliability analysis results of each scale and its dimension.

Scale	Items	Cronbach’s *α*
Chinese Nurse Job Stressors Scale (five dimensions)	35	0.965
Nursing profession and career issues	7	0.915
Workload and time pressure	5	0.892
Resource and environmental problems	3	0.830
Patient care and interaction	11	0.940
Interpersonal relationships and management issues	9	0.936
Decisional Fatigue Scale	9	0.936
Nurse Psychological Capital Questionnaire (four dimensions)	20	0.945
Self‐efficacy	6	0.906
Hope	6	0.913
Resilience	5	0.895
Optimism	3	0.832
Nurse Perceived Organizational Support Scale (two dimensions)	13	0.944
Emotional support	10	0.947
Instrumental support	3	0.834

### 3.3. Common Method Bias

In this study, job stress, DF, PsyCap, and POS were measured as midwives self‐reported methods, and therefore, common method bias may exist. Therefore, we conducted a test for common method bias by Harmam one‐way test method based on the data collection. The results of EFAs showed that the eigenvalues of 12 factors were > 1, and the first factor could only explain 33.647%, which was < 40% critical criterion. Therefore, there was no serious problem of common method bias in the data of this study [41].

### 3.4. Descriptive Statistics and Normality

All variables had absolute values of skewness and kurtosis within acceptable ranges (< |3| for skewness and < |10| for kurtosis) [42], indicating that all variables approximated a normal distribution. Key descriptive statistics are presented in Appendix 3: Job stress total score was 90.45 ± 19.57 (range: 36–138), and mean item score = 2.58 ± 0.5, with the highest mean score in “workload and time pressure” (2.90 ± 0.70) and the lowest in “resource and environmental problems” (2.40 ± 0.73). DF total score was 20.58 ± 5.92 (range: 9–36), and mean item score was 2.29 ± 0.66. PsyCapl total score was 88.83 ± 14.08 (range: 44–120), and mean item score was 4.44 ± 0.70, with the highest score in “self‐efficacy” (4.54 ± 0.80) and the lowest in “hope” (4.37 ± 0.86). POS total score was 43.71 ± 9.98 (range: 14–65) and mean item score was 3.36 ± 0.77, with higher scores in “instrumental support” (3.58 ± 0.82) than “emotional support” (3.30 ± 0.83). Additionally, significant differences in job stress, DF, PsyCap, and POS were observed across various demographic and occupational groups. Detailed results for all comparisons are available in Appendix 4.

### 3.5. Correlation Analysis and Multicollinearity Diagnosis

Pearson correlation analysis revealed significant associations between key variables (Table 2). DF was positively correlated with job stress (*r* = 0.648, *p* < 0.01) and negatively correlated with PsyCap (*r* = −0.471, *p* < 0.01) and POS (*r* = −0.248, *p* < 0.01). PsyCap was negatively correlated with job stress (*r* = ‐ 0.673, *p* < 0.01) and positively correlated with POS (*r* = 0.221, *p* < 0.01). POS was negatively correlated with job stress (*r* = −0.342, *p* < 0.01). To make a diagnosis for any potential multicollinearity among variables, we checked the variance inflation factor (VIF) for each variable; a VIF over 10 is indicative of a multicollinearity problem. Our results show that the VIFs for our explanatory variables did not exceed 2.0 (VIF was 1.322, 1.305, and 1.081 for decisional fatigue, PsyCap, and POS, respectively; tolerance was 0.756, 0.766, and 0.925 for decisional fatigue, PsyCap, and POS, respectively), and we concluded that our sample was devoid of multicollinearity.

**TABLE 2:** The correlation of variable of midwives.

Scale and its dimensions	A	A1	A2	A3	A4	A5	B	C	C1	C2	C3	C4	D	D1	D2
A	1														
A1	0.835^∗∗^	1													
A2	0.786^∗∗^	0.609^∗∗^	1												
A3	0.735^∗∗^	0.563^∗∗^	0.576^∗∗^	1											
A4	0.874^∗∗^	0.642^∗∗^	0.596^∗∗^	0.590^∗∗^	1										
A5	0.844^∗∗^	0.614^∗∗^	0.586^∗∗^	0.556^∗∗^	0.606^∗∗^	1									
B	0.648^∗∗^	0.562^∗∗^	0.512^∗∗^	0.524^∗∗^	0.545^∗∗^	0.536^∗∗^	1								
C	−0.673^∗∗^	−0.572^∗∗^	−0.548^∗∗^	−0.508^∗∗^	−0.575^∗∗^	−0.559^∗∗^	−0.471^∗∗^	1							
C1	−0.552^∗∗^	−0.463^∗∗^	−0.440^∗∗^	−0.410^∗∗^	−0.474^∗∗^	−0.469^∗∗^	−0.391^∗∗^	0.847^∗∗^	1						
C2	−0.571^∗∗^	−0.487^∗∗^	−0.480^∗∗^	−0.423^∗∗^	−0.489^∗∗^	−0.467^∗∗^	−0.383^∗∗^	0.854^∗∗^	0.588^∗∗^	1					
C3	−0.571^∗∗^	−0.484^∗∗^	−0.461^∗∗^	−0.450^∗∗^	−0.485^∗∗^	−0.474^∗∗^	−0.414^∗∗^	0.833^∗∗^	0.598^∗∗^	0.594^∗∗^	1				
C4	−0.540^∗∗^	−0.467^∗∗^	−0.437^∗∗^	−0.406^∗∗^	−0.461^∗∗^	−0.445^∗∗^	−0.381^∗∗^	0.770^∗∗^	0.573^∗∗^	0.561^∗∗^	0.585^∗∗^	1			
D	−0.342^∗∗^	−0.301^∗∗^	−0.267^∗∗^	−0.283^∗∗^	−0.268^∗∗^	−0.303^∗∗^	−0.248^∗∗^	0.221^∗∗^	0.179^∗∗^	0.194^∗∗^	0.166^∗∗^	0.203^∗∗^	1		
D1	−0.341^∗∗^	−0.302^∗∗^	−0.261^∗∗^	−0.280^∗∗^	−0.270^∗∗^	−0.298^∗∗^	−0.247^∗∗^	0.227^∗∗^	0.187^∗∗^	0.198^∗∗^	0.170^∗∗^	0.205^∗∗^	0.981^∗∗^	1	
D2	−0.243^∗∗^	−0.203^∗∗^	−0.205^∗∗^	−0.206^∗∗^	−0.176^∗∗^	−0.227^∗∗^	−0.173^∗∗^	0.132^∗∗^	0.094^∗∗^	0.118^∗∗^	0.102^∗∗^	0.134^∗∗^	0.757^∗∗^	0.616^∗∗^	1

*Note:* A means Chinese Nurse Job Stressors Scale, A1 means nursing profession and career issues, A2 means workload and time pressure, A3 means resource and environmental problems, A4 means patient care and interaction, and A5 means interpersonal relationships and management issues. B means Decisional Fatigue Scale. C means Nurse Psychological Capital Questionnaire, C1 means self‐efficacy, C2 means hope, C3 means resilience, and C4 means optimism. D means Nurse Perceived Organizational Support Scale, D1 means emotional support, and D2 means instrumental support.

^∗^ *p* < 0.05.

^∗∗^ *p* < 0.01.

### 3.6. Mediation Effect Test of Job Stress

**FIGURE 2:**
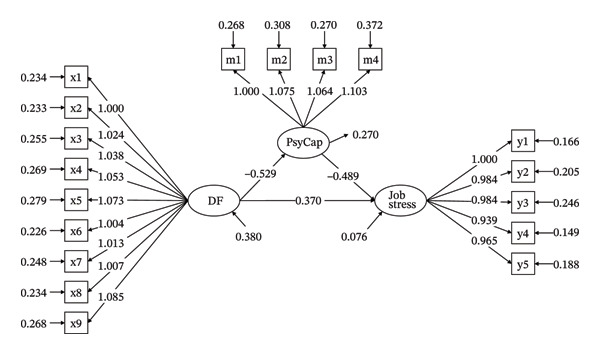
The mediating role of job stress. x1∼x9 means 9‐item of DF, m1∼m4 means 4 dimensions of PsyCap, and y1∼y5 means 5 dimensions of job stress.

SEM was used to test the mediating effect of PsyCap. The model showed good fit: *χ* ^2^/df = 1.353, CFI = 0.997, TLI = 0.996, RMSEA = 0.017, and SRMR = 0.013. DF had a positive direct effect on job stress (*β* = 0.628, *p* < 0.001), thereby confirming H1. After including PsyCap, DF had a negative effect on PsyCap (*β* = −0.529, *p* < 0.001), and PsyCap had a negative effect on job stress (*β* = −0.489, *p* < 0.001). Consequently, H2 and H3 were confirmed. Bootstrap analysis (5000 resamples) confirmed a significant mediating effect of PsyCap: indirect effect = 0.258 (95% CI: 0.223–0.300), accounting for 41.08% of the total effect. Consequently, PsyCap serves as a partial mediator in the relationship between DF and job stress among midwives, thus supporting H4. The direct effect of DF on job stress remained significant (*β* = 0.370, *p* < 0.001) (Figure 2 and Table 3).

**TABLE 3:** Mediating effect of job stress.

Path	Standardized regression coefficient (95% Cl)	SE	*p*	Effect constituting
DF ⟶ PsyCap ⟶ job stress (mediating effect)	0.258 (0.223–0.300)	0.020	< 0.001	41.08%
DF ⟶ job stress (direct effect)	0.370 (0.323–0.419)	0.025	< 0.001	58.92%
Total effect	0.628 (0.578–0.681)	0.026	< 0.001	/

### 3.7. Moderating Role of POS

**FIGURE 3:**
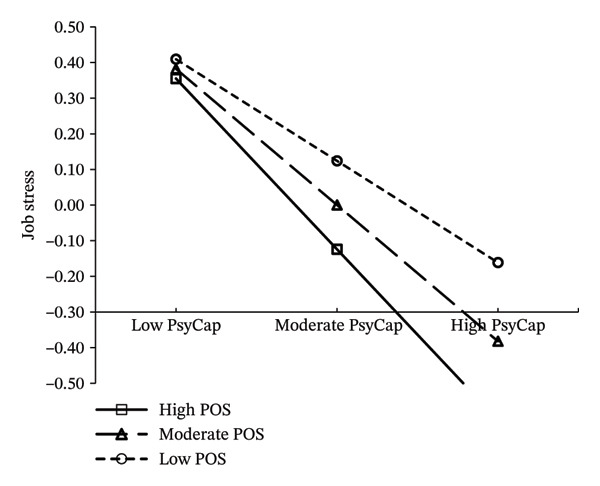
The moderating role of POS.

A LMS tested the moderating effect of POS. The model fit was acceptable (LR = 29.837, df = 1, *p* < 0.001). The interaction term (PsyCap × POS) had a significant negative effect on job stress (*β* = −0.157, *p* < 0.001), indicating that POS moderated the relationship between PsyCap and job stress. Simple slope analysis showed PsyCap had a strong negative effect on job stress (*β* = −0.776, 95% CI: −0.849 to −0.702) at high POS (M + 1SD), and this effect weakened (*β* = −0.461, 95% CI: −0.528 to −0.395) at low POS (M − 1SD) (Figure 3).

### 3.8. Moderated Mediation Effect

**FIGURE 4:**
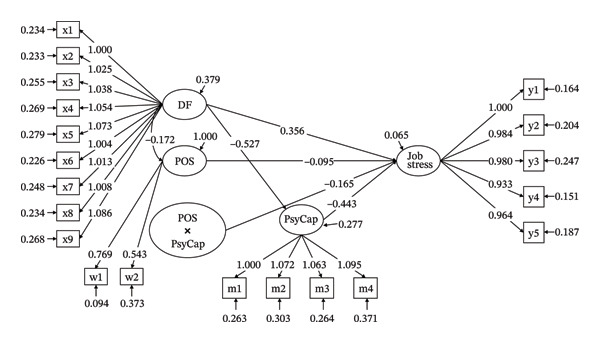
Path diagram of moderated mediation effect test. x1∼x9 means 9‐item of DF, m1∼m4 means 4 dimensions of PsyCap, w1∼w2 means 2 dimensions of POS, and y1∼y5 means 5 dimensions of job stress.

**FIGURE 5:**
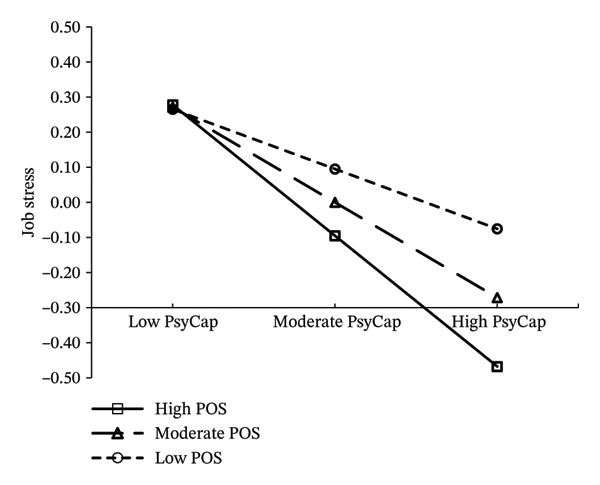
Interaction plot of PsyCap and POS predicting job stress.

The moderated mediation model fit well (LR = 42.217, df = 1, *p* < 0.001), and the results of the moderated mediation effect test show that POS negatively moderated the path between PsyCap and job stress (*γ* = −0.165, *p* < 0.001), indicating a significant moderated mediation effect (Figure 4). We further examined the moderated mediation effect using MODEL CONSTRAINT in Mplus8.3 by testing different levels (above or below one standard deviation) of POS. The results show that POS significantly moderated the indirect effect of DF on job stress via PsyCap: At high POS, the indirect effect was stronger (*γ* = 0.320, *p* < 0.001); at low POS, the indirect effect was weaker (*γ* = 0.147, *p* < 0.001); the difference between high and low POS was significant (*γ* = 0.174, *p* < 0.001), confirming a moderated mediation effect (Figure 5). Thus, H5 was further supported.

## 4. Discussion

This study explored the relationships between DF, PsyCap, POS, and job stress among midwives in Sichuan Province, China. The findings reveal that DF is a significant correlate of job stress, with PsyCap acting as a mediator and POS moderating this pathway. The findings not only confirm the hypothesized relationships but also offer nuanced insights into the mechanisms driving midwives’ workplace stress.

### 4.1. Key Findings in Context

Midwives reported moderate job stress (total score was 90.45 ± 19.57, mean score was 2.58 ± 0.56), with “workload and time pressure” (mean score was 2.90 ± 0.70) as the most pressing concern, which was consistent with previous studies highlighting the tension between declining fertility rates and rising complexity of care for high‐risk pregnancies (e.g., older mothers and multiple pregnancies) [33, 43]. This paradox underscores that reduced birth rates do not equate to reduced workload, instead, the need for enhanced monitoring, emergency preparedness, and personalized care (e.g., doula‐supported deliveries) intensifies time pressures [44]. Second, midwives exhibited high levels of DF (mean score was 2.29 ± 0.66), exceeding those observed in other nursing specialties [45, 46]. This is likely attributable to the unique demands of midwifery: cumulative high‐stakes decisions (e.g., labor progression assessment and complication management) made under time constraints, coupled with the ethical and legal imperatives of ensuring maternal–infant safety [47]. Our results align with neuroscience evidence: repeated decisions deplete prefrontal glucose, impairing executive function and emotional regulation [48, 49]. Younger midwives and those in private hospitals exhibited higher DF, reflecting the role of experience in building decision‐making resilience and the potential resource constraints in nonpublic settings [50]. Third, PsyCap was moderately high (mean score was 4.44 ± 0.70), with “self‐efficacy” rooted in clinical competence scoring highest (mean score was 4.54 ± 0.80) and “hope” lowest (mean score was 4.37 ± 0.86). This mirrors findings that clinical competence was fostered by tertiary hospitals’ complex cases and midwives’ independent decision‐making in physiological births, boosting self‐efficacy, while limited career progression and chronic stress may dampen hope [51, 52]. The negative correlation between DF and PsyCap (*r* = −0.471, *p* < 0.01) aligns with research showing that repeated decision‐making erodes positive psychological resources [53]. Additionally, POS was also moderate, with stronger “instrumental support” (e.g., equipment, training, and mean score was 3.58 ± 0.82) than “emotional support” (e.g., recognition, communication, and mean score was 3.30 ± 0.83), mirroring institutional priorities in health care where material resources are often prioritized over psychological well‐being [54, 55]. Higher POS among older, more experienced midwives suggests that organizational investments in long‐term staff may enhance perceived support [56].

### 4.2. Demographic Variations

Job stress varied significantly by age, ethnicity, hospital type/level, employment status, seniority, and night‐shift frequency. Younger, less‐experienced midwives reported higher stress, consistent with reduced clinical confidence. DF was elevated in males, younger midwives, those in general/private hospitals, and managerial roles, reflecting role‐specific pressures. PsyCap was lower in younger midwives and private‐hospital staff, correlating with limited autonomy and development opportunities. POS disparities emerged across age, gender, education, hospital level, and income. Junior staff and those in lower‐tier hospitals perceived less support, exacerbating stress. These findings underscore the need for tailored interventions addressing demographic inequities.

### 4.3. Mediating/Moderating Effects

DF directly increased job stress (*β* = 0.628, *p* < 0.001) and reduced PsyCap (*r* = −0.471, *p* < 0.01). PsyCap partially mediated the DF⟶ job stress pathway (indirect effect: 0.258, 95% CI [0.223, 0.300]), accounting for 41.08% of the total effect. This aligns with the JD‐R theory that DF (a job demand) depletes cognitive resources, while PsyCap (a job resource) buffers job stress by fostering resilience, self‐efficacy, and solution‐focused coping.

POS functioned as both a direct resource and a moderator. Our moderated mediation analysis revealed that higher POS strengthened PsyCap’s job stress‐buffering effect (high POS: *β* = −0.776, *p* < 0.001 vs. low POS: *β* = −0.461, *p* < 0.001). This aligns with the JD‐R model, where organizational resources (POS) enhance the efficacy of personal resources (PsyCap) in mitigating demands (DF) [57, 58]. Specifically, midwives with high POS likely perceive stronger institutional backing because they may perceive institutional backing as a safety net, enabling them to leverage PsyCap more effectively to counteract job stress.

### 4.4. Implications for Theory and Practice

#### 4.4.1. Theoretical Implications

This study advances an understanding of midwifery stress by integrating DF, PsyCap, and POS within a unified JD‐R framework. By demonstrating that DF acts as a key demand, PsyCap as a mediating resource, and POS as a moderating resource, we extend the JD‐R model’s applicability to high‐stakes clinical contexts. This framework clarifies how (via PsyCap) and under what conditions (under high POS) DF impacts job stress, addressing gaps in the literature that often focus on isolated stressors or resources. Specifically, identifying PsyCap as a partial mediator expands the understanding of the health impairment process, showing that the adverse effects of DF are not only direct but also indirect, through the depletion of positive psychological resources. Additionally, the moderating role of POS underscores the importance of contextual factors in shaping the effectiveness of personal resources, suggesting that organizational support can amplify the stress‐buffering effects of PsyCap. This extends previous research by showing that the impact of individual resources on stress is not static but is contingent on organizational context, providing a more nuanced understanding of stress mechanisms in midwifery.

#### 4.4.2. Practical Implications for Nurse Leaders

Clarifying the complex pathway linking DF, PsyCap, and POS is critical for nurse leaders to formulate targeted, feasible intervention measures. Combined with classic implementation theories including the Capability–Opportunity–Motivation–Behavior (COM‐B) model and Behavior Change Wheel (BCW) [59–61], the present findings can guide multilevel optimization of midwifery management, resolve practical implementation barriers, and translate theoretical evidence into daily clinical management practice. At the capability level, nurse leaders should prioritize interventions to enhance midwives’ cognitive and psychological capacity to cope with DF and stress. This includes implementing regular cognitive training programs focused on decision‐making efficiency (e.g., algorithmic labor progression charts and clinical decision‐support tools) to reduce cognitive load [62, 63]. Additionally, resilience‐building workshops (e.g., mindfulness‐based stress reduction and cognitive‐behavioral training) and mentorship programs pairing junior and senior midwives can strengthen PsyCap, particularly hope and resilience through knowledge sharing, clinical confidence building, and emotional guidance [64]. Nurse leaders should also ensure that midwives have access to ongoing professional development opportunities, as enhanced clinical competence directly boosts self‐efficacy, a key component of PsyCap. At the opportunity level, nurse leaders must address organizational barriers exacerbating DF and stress by optimizing staffing allocation to reduce workload and time pressure, especially during peak periods (e.g., high‐volume labor and delivery shifts) [65], and implementing flexible shift models (e.g., paired shifts and scheduled cognitive breaks) to prevent cumulative DF [66]. They should also improve the clinical environment with adequate instrumental support (advanced monitoring equipment, streamlined documentation, and rest spaces) and reduce administrative burden (e.g., simplified reporting) to free up time for clinical work and self‐care, further mitigating DF [67]. At the motivation level, nurse leaders should enhance emotional support and incentives to boost midwives’ professional identity and engagement, including regular feedback to recognize contributions, formal recognition programs (e.g., clinical excellence awards), clear career progression pathways to foster hope, and equitable support resources for vulnerable groups (especially for junior midwives and private hospital staff) to ensure adequate organizational backing [60, 68]. Meanwhile, nurse leaders should fully anticipate common implementation barriers, such as tight clinical manpower, limited management funds, heavy daily work tasks, and low individual participation willingness. Corresponding solutions include phased promotion of intervention measures, simplified operation of management schemes, regular communication and feedback, and hierarchical management for junior and senior midwives, so as to reduce implementation resistance and ensure the long‐term effectiveness of relevant optimization strategies.

### 4.5. Strengths and Limitations

Despite its contributions, this study has limitations. First, the regional sampling restricted to Sichuan Province limits the generalizability of the findings, multicenter studies across diverse healthcare systems are needed to confirm the external validity of our results. Second, the general information questionnaire did not undergo formal psychometric validation. Nevertheless, content validity was ensured through expert review, face validity was tested during a pilot study with subsequent item revisions, and standardized training of survey administrators, as well as post hoc logical data cleaning were implemented to enhance data quality. In addition, regarding the measurement instruments, we deliberately used well‐validated general scales because it is essential to first capture the full picture of midwives’ job stress status and occupational characteristics. Future studies should develop or adopt more contemporary, midwife‐specific instruments to capture the nuances of this unique profession. Third, although Harman’s single‐factor test indicated minimal common‐method variance, self‐reported data remain susceptible to response bias; future studies may consider supplementing subjective measures with objective biomarkers (e.g., cortisol for stress and actigraphy for fatigue). Finally, although we used SEM to test directional hypotheses based on theoretical assumptions, the cross‐sectional design precludes causal inference. The observed associations should therefore be interpreted as statistical relationships rather than causal effects. Future research should employ longitudinal designs to establish temporal relationships among these variables. Moreover, randomized controlled trials testing interventions targeting PsyCap and POS are urgently needed, particularly in low‐resource settings. Despite these limitations, this study provides meaningful insights into the mechanisms linking DF to job stress among midwives.

## 5. Conclusion

This study demonstrates that DF significantly exacerbates midwives’ job stress, with PsyCap acting as a critical partial mediator accounting for 41.08% of the total effect, while POS moderates the stress pathway by reinforcing the protective function of PsyCap. These findings validate the utility of the JD‐R model in understanding midwives’ occupational stress and highlight the synergistic importance of addressing decision demands, fostering positive psychological resources, and enhancing organizational support. In low‐fertility eras with rising maternal risks, interventions targeting DF (e.g., workload management), PsyCap (e.g., resilience training), and POS (e.g., emotional support initiatives) are essential to sustain midwifery workforce stability and quality of care. Longitudinal and interventional research conducted in diverse medical settings is recommended in future studies.

## Author Contributions

Xianying Lu, Yimin Zhang, and Zhirong Ren contributed equally to this work. This idea was thought by Xianying Lu, Yimin Zhang, and Zhirong Ren. This primary article was written by Xianying Lu, Yimin Zhang, and Zhirong Ren. Jing Gao and Chaoming Hou were responsible for revising this article. Xianying Lu and Yimin Zhang performed the initial screening. Xianying Lu and Zhirong Ren extracted and interpreted the data. Xianying Lu and Zhirong Ren performed the quality assessment. Xinyu Chen performed the statistical analysis. Dingxi Bai was responsible for the production and organization of the figures and tables.

## Funding

This work was supported by the Sichuan Medical Law Research Center (No. 23YFYB008).

## Disclosure

Xianying Lu, Yimin Zhang, and Zhirong Ren are co‐first authors. All of the listed authors have actively participated in the study and have both seen and approved the article.

## Ethics Statement

Ethical approval was obtained from the Ethics Committee of the Fifth People’s Hospital of Chengdu (No: 2024‐058‐01), and all patients provided consent to participate in this study.

## Consent

Please see the Ethics Statement.

## Conflicts of Interest

The authors declare no conflicts of interest.

## Supporting Information

Additional supporting information can be found online in the Supporting Information section.

## Supporting information


**Supporting Information** Appendix 1—Appendix 4.

## Data Availability

The data that support the findings of this study are available from the corresponding authors upon reasonable request.
