# Supplementary material for: The Effect of Decision Fatigue on Job Stress in Midwives: The Mediating Role of Psychological Capital and the Moderating Effect of Perceived Organizational Support
Source: J Nurs Manag. 2026 May 29;2026:4433124. doi: 10.1155/jonm/4433124 (PMC13239171; doi:10.1155/jonm/4433124)
Supplement: Supplementary file 1 — Supporting Information Appendix 1—Appendix 4. [file JONM-2026-4433124-s001.docx]

# Appendix 1: Sampling method

A multistage stratified cluster sampling approach was used:

Stage 1 (city stratification): Sichuan Province was divided into five economic zones, including Chengdu Plain Economic Zone (8 cities/prefectures of Chengdu, Deyang, Mianyang, Leshan, Meishan, Ziyang, Suining, Ya'an), Southern Sichuan Economic Zone (4 cities/prefectures of Zigong, Luzhou, Neijiang, Yibin)), Northeast Sichuan Economic Zone (5 cities/prefectures of Guangyuan, Nanchong, Guang'an, Dazhou, Bazhong), Panxi Economic Zone (2 cities/prefectures of Panzhihua, Liangshan Yi Autonomous Prefecture), and West Sichuan Economic Zone (2 prefectures of Ganzi Tibetan Autonomous Prefecture, Aba Tibetan and Qiang Autonomous Prefecture). The number of cities sampled from each zone was proportional to their 2023 GDP share: Chengdu Plain Economic Zone (62%, 8 cities), Southern Sichuan Economic Zone (16%, 3 cities), Northeastern Sichuan Economic Zone (14%, 3 cities), Panxi Economic Zone (6%, 1 city), and Western Sichuan Ecological Economic Zone (2%, 1 city). A total of 16 cities/autonomous prefectures were selected, including Chengdu Plain Economic Zone (Chengdu, Deyang, Mianyang, Leshan, Meishan, Ziyang, Suining, Ya’an ), Southern Sichuan Economic Zone (Yibin, Luzhou, Zigong), Northeast Sichuan Economic Zone (Guangyuan, Dazhou, Nanchong), Panxi Economic Zone (Panzhihua), and West Sichuan Economic Zone (Ganzi Tibetan Autonomous Prefecture).

Stage 2 (hospital stratification): The sampling frame for hospitals was established based on the official "Sichuan Province Baby-Friendly Hospital List (current as of April 2024)". Analysis of this registry revealed the provincial ratio of primary, secondary, tertiary hospitals to be approximately 2:5:3. Consequently, the target sample size for hospitals within the previously sampled cities/prefectures was stratified according to this established provincial ratio (Level primary, secondary, tertiary = 2:5:3). Furthermore, within each hospital level primary, secondary, tertiary), sampling quotas were allocated proportionally based on the documented provincial distribution of Class A and Class B hospitals. Finally, 146 hospitals were included: 45 tertiary (26 Grade A, 19 Grade B), 79 secondary (46 Grade A, 33 Grade B), and 22 primary (20 Grade A, 2 Grade B).

Third stage (midwife selection): All eligible midwives from the 146 selected hospitals were invited to participate.

# Appendix 2: Demographic and occupational characteristics

| Variable | group | N (%) | |
| --- | --- | --- | --- |
| age | ≤25 | 160 (12.7) | |
|  | 26-30 | 312 (24.7) | |
|  | 31-40 | 583 (46.2) | |
|  | 41-50 | 169 (13.4) | |
|  | ＞50 | 39 (3.1) | |
| gender | male | 28 (2.2) | |
|  | female | 1235 (97.8) | |
| ethnicity | Han | 1200 (95) | |
|  | Tibetan | 34 (2.7) | |
|  | Qiang | 10 (0.8) | |
|  | Yi | 11 (0.9) | |
|  | Hui | 8 (0.6) | |
| educational level | technical secondary school | 12 (1) | |
|  | junior college | 248 (19.6) | |
|  | bachelor's degree | 988 (78.2) | |
|  | master's degree | 15 (1.2) | |
| hospital type | general hospital | 924 (73.2) | |
|  | specialty hospital | 339 (26.8) | |
| hospital nature | public hospital | 1005 (79.6) | |
|  | private hospital | 258 (20.4) | |
| hospital grade | primary A level | 41 (3.2) | |
|  | Primary B level | 5 (0.4) | |
|  | secondary A level | 292 (23.1) | |
|  | secondary B level | 134 (10.6) | |
|  | tertiary A level | 487 (38.6) | |
|  | tertiary B level | 304 (24.1) | |
| employment type | bianzhi | 240 (19) | |
|  | contract-based | 985 (78) | |
|  | personnel agent | 38 (3) | |
| work experience | ＜5 years | 214 (16.9) | |
|  | 5-10 years | 436 (34.5) | |
|  | 11-20 years | 439 (34.8) | |
|  | 21-30 years | 136 (10.8) | |
|  | ＞30 years | 38 (3) | |
| professional title | staff nurse | 183 (14.5) | |
|  | primary nurse | 387 (30.6) | |
|  | nurse-in-charge | 594 (47) | |
|  | associate chief nurse | 93 (7.4) | |
|  | chief nurse | 6 (0.5) | |
| professional position | none | 1140 (90.3) | |
|  | deputy midwife (deputy head nurse) | 58 (4.6) | |
|  | midwives (head nurses) and above | 65 (5.1) | |
| monthly night shifts | 0 | 257 (20.3) | |
|  | 1-4 | 264 (20.9) | |
|  | 5-9 | 487 (38.6) | |
|  | ＞9 | 255 (20.2) | |
| monthly income | ＜5000 yuan | 405 | 32.1 |
|  | 5000-7999 yuan | 652 | 51.6 |
|  | ≥8000 yuan | 206 | 16.3 |
| marital status | unmarried | 312 | 24.7 |
|  | married | 919 | 72.8 |
|  | divorced | 31 | 2.5 |
|  | widowed | 1 | 0.1 |
| fertility status | none | 398 | 31.5 |
|  | one child | 568 | 45 |
|  | two children | 291 | 23 |
|  | three or more children | 6 | 0.5 |

| Scale and its dimensions | items | score _min_ | score _max_ | score _total_（$\bar{x}\pm s$） | score _mean_（$\bar{x}\pm s$） | skewness | kurtosis |
| --- | --- | --- | --- | --- | --- | --- | --- |
| Chinese Nurse Job Stressors Scale | 35 | 36 | 138 | 90.45±19.57 | 2.58±0.56 | —0.114 | —0.407 |
| nursing profession and career issues | 7 | 7 | 28 | 19.21±4.73 | 2.74±0.68 | —0.186 | —0.593 |
| workload and time pressure | 5 | 5 | 20 | 14.49±3.49 | 2.90±0.70 | —0.358 | —0.539 |
| resource and environmental problems | 3 | 3 | 12 | 7.20±2.18 | 2.40±0.73 | 0.035 | —0.542 |
| patient care and interaction | 11 | 11 | 44 | 27.71±7.00 | 2.52±0.64 | —0.041 | —0.432 |
| interpersonal relationships and manage ment issues | 9 | 9 | 36 | 21.85±6.10 | 2.43±0.68 | —0.033 | —0.535 |
| Decisional Fatigue Scale | 9 | 9 | 36 | 20.58±5.92 | 2.29±0.66 | 0.125 | -0.532 |
| Nurse Psychological Capital Questionnaire | 20 | 44 | 120 | 88.83±14.08 | 4.44±0.70 | —0.156 | —0.348 |
| self-efficacy | 6 | 13 | 36 | 27.23±4.82 | 4.54±0.80 | —0.203 | —0.476 |
| hope | 6 | 9 | 36 | 26.21±5.17 | 4.37±0.86 | —0.214 | —0.325 |
| resilience | 5 | 10 | 30 | 22.24±4.17 | 4.45±0.83 | —0.226 | —0.400 |
| optimism | 3 | 3 | 18 | 13.14±2.73 | 4.38±0.91 | —0.230 | —0.377 |
| Nurse Perceived Organizational Support Scale | 13 | 14 | 65 | 43.71±9.98 | 3.36±0.77 | —0.166 | —0.420 |
| emotional support | 10 | 10 | 50 | 32.98±8.28 | 3.30±0.83 | —0.160 | —0.463 |
| instrumental support | 3 | 3 | 15 | 10.73±2.45 | 3.58±0.82 | —0.335 | —0.142 |

# Appendix 3: Descriptive statistics

# Appendix 4: Group differences in key variables

Age: Significant differences were found across all age groups (≤25, 26-30, 31-40, 41-50, >50 years) for all key variables (Job Stress, Decision Fatigue, Psychological Capital, and Perceived Organizational Support; all p<0.05). Midwives aged ≤25 years consistently reported significantly higher levels of job stress and decision fatigue, and significantly lower levels of psychological capital and perceived organizational support compared to all older age groups. Psychological capital and perceived organizational support generally increased with age.

Gender: Male midwives (n=28) reported significantly higher decision fatigue (t=3.023, p=0.005) but significantly lower perceived organizational support (t=-3.945, p<0.001) and emotional support (t=-4.288, p<0.001) compared to female midwives (n=1235). No significant gender differences were found for job stress or psychological capital.

Hospital Type: Midwives in specialist hospitals (n=339) reported significantly lower decision fatigue (t=2.235, p=0.026) but significantly higher perceived organizational support (t=-3.550, p<0.001), emotional support (t=-3.300, p=0.001), and instrumental support (t=-3.178, p=0.002) compared to those in general hospitals (n=924). No significant differences were found for job stress or psychological capital.

Hospital nature: Midwives in private hospitals (n=258) reported significantly higher levels of job stress (t=-9.132, p<0.001) across all its subscales and decision fatigue (t=-4.118, p<0.001), but significantly lower levels of psychological capital (t=5.569, p<0.001) across all its subscales and perceived organizational support (t=2.592, p=0.010) / emotional support (t=2.707, p=0.007) compared to those in public hospitals (n=1005). No difference was found for instrumental support.

Education Level: Significant differences were found for perceived organizational support and its subscales (p<0.001). Midwives with bachelor's or master's degrees reported significantly higher levels of perceived organizational support and its subscales compared to those with diplomas or associate degrees. No significant differences were found for job stress, decision fatigue, or psychological capital across education levels.

Employment Type: Midwives with formal establishment ("Bianzhi", n=240) reported significantly higher job stress (F=3.409, p=0.033), workload and time pressure (F=3.797, p=0.023), interpersonal relationships and manage ment issues (F=3.024, p=0.049), and perceived organizational support (F=4.993, p=0.007) / its subscales compared to those on contract-based (n=985). No differences were found for decision fatigue or psychological capital.

Work Experience: Significant differences were found for job stress (F=3.461, p=0.008) and several subscales, psychological capital (F=2.386, p=0.049), and perceived organizational support and its subscales (all p<0.001). Generally, midwives with less than 5 years experience reported higher job stress but lower perceived organizational support compared to more experienced groups. Psychological capital was lower in the <5 years group compared to groups with 5+ years.

Professional Title: Significant differences were found only for perceived organizational support and its subscales (p<0.001). Staff Nurse and Primary Nurse reported significantly lower levels compared to Nurse-in-Charge, Deputy Chief Nurses, and Chief Nurses. No differences were found for job stress, decision fatigue, or psychological capital.

Monthly Income: Significant differences were found for perceived organizational support and its subscales (p<0.001). Midwives earning ≥8000 yuan reported the highest levels, followed by those earning 5000-7999 yuan, with those earning <5000 yuan reporting the lowest levels. No differences were found for job stress, decision fatigue, or psychological capital.

Other Variables (Summarized): Significant differences related to Ethnicity were found for job stress and specific subscales (Han vs. Tibetan differences noted). Hospital Grade differences were found for job stress (specific subscales) and perceived organizational support. Position differences were only found for decision fatigue (Deputy Head Midwives higher than no position). Night Shifts differences were found for job stress (all subscales higher in >9 shifts group) and perceived organizational support (higher in 5-9 and >9 shifts groups). Marital Status differences were only found for perceived organizational support, but post-hoc tests were not feasible due to small group sizes (widowed n=1).

## The difference test of the variables in different ages (N=1263)

| Scale and its dimensions | group | n | score _mean_（$\bar{x}\pm s$） | *F* value | *P* value | multiple comparisons |
| --- | --- | --- | --- | --- | --- | --- |
| Chinese Nurse Job Stressors Scale | ≤25 | 160 | 2.76±0.57 | 7.201 | 0.000 | 1＞2；1＞3；1＞4；  1＞5；2＞5；3＞5 |
|  | 26-30 | 312 | 2.6±0.53 |  |  |  |
|  | 31-40 | 583 | 2.56±0.55 |  |  |  |
|  | 41-50 | 169 | 2.51±0.57 |  |  |  |
|  | ＞50 | 39 | 2.33±0.73 |  |  |  |
| nursing profession and career issues | ≤25 | 160 | 2.95±0.61 | 6.301 | 0.000 | 1＞2；1＞3；1＞4；  1＞5；2＞5；4＜2 |
|  | 26-30 | 312 | 2.75±0.67 |  |  |  |
|  | 31-40 | 583 | 2.73±0.66 |  |  |  |
|  | 41-50 | 169 | 2.62±0.69 |  |  |  |
|  | ＞50 | 39 | 2.52±0.91 |  |  |  |
| workload and time pressure | ≤25 | 160 | 3.07±0.65 | 3.965 | 0.003 | 1＞2；1＞3；1＞4；1＞5；2＞5 |
|  | 26-30 | 312 | 2.9±0.68 |  |  |  |
|  | 31-40 | 583 | 2.88±0.7 |  |  |  |
|  | 41-50 | 169 | 2.85±0.69 |  |  |  |
|  | ＞50 | 39 | 2.66±0.89 |  |  |  |
| resource and environmental problems | ≤25 | 160 | 2.55±0.72 | 4.721 | 0.001 | 1＞3；1＞4；1＞5；2＞5；3＞5；4＞5 |
|  | 26-30 | 312 | 2.41±0.7 |  |  |  |
|  | 31-40 | 583 | 2.39±0.73 |  |  |  |
|  | 41-50 | 169 | 2.36±0.76 |  |  |  |
|  | ＞50 | 39 | 2.01±0.71 |  |  |  |
| patient care and interaction | ≤25 | 160 | 2.69±0.66 | 6.739 | 0.000 | 1＞3；1＞4；1＞5；2＞4；2＞5；3＞5 |
|  | 26-30 | 312 | 2.58±0.6 |  |  |  |
|  | 31-40 | 583 | 2.49±0.63 |  |  |  |
|  | 41-50 | 169 | 2.41±0.63 |  |  |  |
|  | ＞50 | 39 | 2.25±0.78 |  |  |  |
| interpersonal relationships and manage ment issues | ≤25 | 160 | 2.61±0.71 | 4.247 | 0.002 | 1＞2；1＞3；1＞4；1＞5 |
|  | 26-30 | 312 | 2.42±0.67 |  |  |  |
|  | 31-40 | 583 | 2.4±0.66 |  |  |  |
|  | 41-50 | 169 | 2.42±0.65 |  |  |  |
|  | ＞50 | 39 | 2.2±0.86 |  |  |  |
| Decisional Fatigue Scale | ≤25 | 160 | 2.45±0.64 | 3.821 | 0.004 | 1＞2；1＞3；1＞4；1＞5 |
|  | 26-30 | 312 | 2.28±0.65 |  |  |  |
|  | 31-40 | 583 | 2.28±0.66 |  |  |  |
|  | 41-50 | 169 | 2.22±0.65 |  |  |  |
|  | ＞50 | 39 | 2.11±0.72 |  |  |  |
| Nurse Psychological Capital Questionnaire | ≤25 | 160 | 4.22±0.74 | 5.322 | 0.000 | 1＜2；1＜3；1＜4；1＜5 |
|  | 26-30 | 312 | 4.45±0.72 |  |  |  |
|  | 31-40 | 583 | 4.47±0.68 |  |  |  |
|  | 41-50 | 169 | 4.48±0.66 |  |  |  |
|  | ＞50 | 39 | 4.64±0.76 |  |  |  |
| self-efficacy | ≤25 | 160 | 4.33±0.87 | 3.801 | 0.004 | 1＜2；1＜3；1＜4；1＜5 |
|  | 26-30 | 312 | 4.53±0.85 |  |  |  |
|  | 31-40 | 583 | 4.58±0.77 |  |  |  |
|  | 41-50 | 169 | 4.57±0.74 |  |  |  |
|  | ＞50 | 39 | 4.75±0.81 |  |  |  |
| hope | ≤25 | 160 | 4.14±0.91 | 3.573 | 0.007 | 1＜2；1＜3；1＜4；1＜5 |
|  | 26-30 | 312 | 4.38±0.85 |  |  |  |
|  | 31-40 | 583 | 4.4±0.86 |  |  |  |
|  | 41-50 | 169 | 4.41±0.83 |  |  |  |
|  | ＞50 | 39 | 4.57±0.91 |  |  |  |
| resilience | ≤25 | 160 | 4.22±0.86 | 3.82 | 0.004 | 1＜2；1＜3；1＜4；1＜5 |
|  | 26-30 | 312 | 4.49±0.82 |  |  |  |
|  | 31-40 | 583 | 4.48±0.82 |  |  |  |
|  | 41-50 | 169 | 4.44±0.83 |  |  |  |
|  | ＞50 | 39 | 4.63±0.89 |  |  |  |
| optimism | ≤25 | 160 | 4.13±0.97 | 4.233 | 0.002 | 1＜2；1＜3；1＜4；1＜5 |
|  | 26-30 | 312 | 4.39±0.91 |  |  |  |
|  | 31-40 | 583 | 4.4±0.91 |  |  |  |
|  | 41-50 | 169 | 4.48±0.86 |  |  |  |
|  | ＞50 | 39 | 4.59±0.82 |  |  |  |
| Nurse Perceived Organizational Support Scale | ≤25 | 160 | 2.96±0.77 | 16.611 | 0.000 | 1＜2；1＜3；1＜4；1＜5；2＜3；2＜4 |
|  | 26-30 | 312 | 3.29±0.74 |  |  |  |
|  | 31-40 | 583 | 3.47±0.78 |  |  |  |
|  | 41-50 | 169 | 3.5±0.67 |  |  |  |
|  | ＞50 | 39 | 3.41±0.65 |  |  |  |
| emotional support | ≤25 | 160 | 2.87±0.82 | 16.031 | 0.000 | 1＜2；1＜3；1＜4；1＜5；2＜3；2＜4 |
|  | 26-30 | 312 | 3.22±0.81 |  |  |  |
|  | 31-40 | 583 | 3.4±0.84 |  |  |  |
|  | 41-50 | 169 | 3.46±0.7 |  |  |  |
|  | ＞50 | 39 | 3.38±0.74 |  |  |  |
| instrumental support | ≤25 | 160 | 3.26±0.83 | 9.757 | 0.000 | 1＜2；1＜3；1＜4；2＜3 |
|  | 26-30 | 312 | 3.5±0.79 |  |  |  |
|  | 31-40 | 583 | 3.69±0.83 |  |  |  |
|  | 41-50 | 169 | 3.65±0.79 |  |  |  |
|  | ＞50 | 39 | 3.5±0.64 |  |  |  |
| Note：1 means ≤25 age, 2 means 26-30 age, 3 means 31-40 age, 4 means 41-50 age, 5 means ＞50 age. | | | | | | |

## The difference test of the variables in different gender (N=1263)

| Scale and its dimensions | group | n | score _mean_（$\bar{x}\pm s$） | *F* value | *P* value |
| --- | --- | --- | --- | --- | --- |
| Chinese Nurse Job Stressors Scale | male | 28 | 2.73±0.46 | 1.39 | 0.165 |
|  | female | 1235 | 2.58±0.56 |  |  |
| nursing profession and career issues | male | 28 | 2.90±0.63 | 1.263 | 0.207 |
|  | female | 1235 | 2.74±0.68 |  |  |
| workload and time pressure | male | 28 | 2.99±0.69 | 0.731 | 0.465 |
|  | female | 1235 | 2.90±0.70 |  |  |
| resource and environmental problems | male | 28 | 2.54±0.67 | 1.082 | 0.279 |
|  | female | 1235 | 2.40±0.73 |  |  |
| patient care and interaction | male | 28 | 2.71±0.51 | 2.012 | 0.054 |
|  | female | 1235 | 2.51±0.64 |  |  |
| interpersonal relationships and manage ment issues | male | 28 | 2.53±0.63 | 0.821 | 0.412 |
|  | female | 1235 | 2.43±0.68 |  |  |
| Decisional Fatigue Scale | male | 28 | 2.55±0.46 | 3.023 | 0.005 |
|  | female | 1235 | 2.28±0.66 |  |  |
| Nurse Psychological Capital Questionnaire | male | 28 | 4.36±0.58 | -0.64 | 0.523 |
|  | female | 1235 | 4.44±0.71 |  |  |
| self-efficacy | male | 28 | 4.36±0.93 | -1.208 | 0.227 |
|  | female | 1235 | 4.54±0.80 |  |  |
| hope | male | 28 | 4.52±0.75 | 0.965 | 0.335 |
|  | female | 1235 | 4.36±0.86 |  |  |
| resilience | male | 28 | 4.31±0.55 | -1.295 | 0.205 |
|  | female | 1235 | 4.45±0.84 |  |  |
| optimism | male | 28 | 4.09±0.75 | -1.683 | 0.093 |
|  | female | 1235 | 4.38±0.91 |  |  |
| Nurse Perceived Organizational Support Scale | male | 28 | 2.80±0.58 | -3.945 | 0.000 |
|  | female | 1235 | 3.38±0.77 |  |  |
| emotional support | male | 28 | 2.64±0.71 | -4.288 | 0.000 |
|  | female | 1235 | 3.31±0.82 |  |  |
| instrumental support | male | 28 | 3.33±0.63 | -1.586 | 0.113 |
|  | female | 1235 | 3.58±0.82 |  |  |

## The difference test of the variables in different ethnicity (N=1263)

| Scale and its dimensions | group | n | score _mean_（$\bar{x}\pm s$） | *F* value | *P* value | multiple comparisons |
| --- | --- | --- | --- | --- | --- | --- |
| Chinese Nurse Job Stressors Scale | Han | 1200 | 2.58±0.56 | 3.214 | 0.012 | 1＜2；1＞5；2＞4；2＞5 |
|  | Tibetan | 34 | 2.84±0.44 |  |  |  |
|  | Qiang | 10 | 2.59±0.52 |  |  |  |
|  | Yi | 11 | 2.45±0.42 |  |  |  |
|  | Hui | 8 | 2.14±0.61 |  |  |  |
| nursing profession and career issues | Han | 1200 | 2.74±0.68 | 3.416 | 0.009 | 1＜2；1＞5；2＞5；3＞5；4＞5 |
|  | Tibetan | 34 | 2.98±0.48 |  |  |  |
|  | Qiang | 10 | 2.96±0.7 |  |  |  |
|  | Yi | 11 | 2.69±0.5 |  |  |  |
|  | Hui | 8 | 2.05±0.77 |  |  |  |
| workload and time pressure | Han | 1200 | 2.89±0.7 | 1.626 | 0.165 | / |
|  | Tibetan | 34 | 3.17±0.63 |  |  |  |
|  | Qiang | 10 | 2.9±0.54 |  |  |  |
|  | Yi | 11 | 2.8±0.64 |  |  |  |
|  | Hui | 8 | 2.65±0.69 |  |  |  |
| resource and environmental problems | Han | 1200 | 2.4±0.73 | 2.084 | 0.081 | / |
|  | Tibetan | 34 | 2.73±0.61 |  |  |  |
|  | Qiang | 10 | 2.23±0.59 |  |  |  |
|  | Yi | 11 | 2.21±0.52 |  |  |  |
|  | Hui | 8 | 2.29±1 |  |  |  |
| patient care and interaction | Han | 1200 | 2.52±0.64 | 2.502 | 0.041 | 1＜2；1＞5；2＞5 |
|  | Tibetan | 34 | 2.77±0.57 |  |  |  |
|  | Qiang | 10 | 2.44±0.64 |  |  |  |
|  | Yi | 11 | 2.4±0.54 |  |  |  |
|  | Hui | 8 | 2.06±0.5 |  |  |  |
| interpersonal relationships and manage ment issues | Han | 1200 | 2.43±0.68 | 2.256 | 0.061 | / |
|  | Tibetan | 34 | 2.67±0.65 |  |  |  |
|  | Qiang | 10 | 2.43±0.59 |  |  |  |
|  | Yi | 11 | 2.23±0.52 |  |  |  |
|  | Hui | 8 | 1.96±1.02 |  |  |  |
| Decisional Fatigue Scale | Han | 1200 | 2.28±0.66 | 1.386 | 0.236 | / |
|  | Tibetan | 34 | 2.47±0.55 |  |  |  |
|  | Qiang | 10 | 2.12±0.72 |  |  |  |
|  | Yi | 11 | 2.45±0.5 |  |  |  |
|  | Hui | 8 | 2±0.71 |  |  |  |
| Nurse Psychological Capital Questionnaire | Han | 1200 | 4.44±0.71 | 1.265 | 0.282 | / |
|  | Tibetan | 34 | 4.46±0.46 |  |  |  |
|  | Qiang | 10 | 4.44±0.72 |  |  |  |
|  | Yi | 11 | 4.55±0.51 |  |  |  |
|  | Hui | 8 | 4.98±0.64 |  |  |  |
| self-efficacy | Han | 1200 | 4.53±0.81 | 2.306 | 0.056 | / |
|  | Tibetan | 34 | 4.48±0.68 |  |  |  |
|  | Qiang | 10 | 4.78±0.63 |  |  |  |
|  | Yi | 11 | 4.71±0.72 |  |  |  |
|  | Hui | 8 | 5.31±0.56 |  |  |  |
| hope | Han | 1200 | 4.36±0.87 | 0.307 | 0.873 | / |
|  | Tibetan | 34 | 4.51±0.62 |  |  |  |
|  | Qiang | 10 | 4.33±0.97 |  |  |  |
|  | Yi | 11 | 4.33±0.61 |  |  |  |
|  | Hui | 8 | 4.5±0.87 |  |  |  |
| resilience | Han | 1200 | 4.44±0.84 | 1.422 | 0.224 | / |
|  | Tibetan | 34 | 4.44±0.62 |  |  |  |
|  | Qiang | 10 | 4.34±0.79 |  |  |  |
|  | Yi | 11 | 4.56±0.7 |  |  |  |
|  | Hui | 8 | 5.13±0.58 |  |  |  |
| optimism | Han | 1200 | 4.38±0.91 | 1.465 | 0.21 | / |
|  | Tibetan | 34 | 4.35±0.83 |  |  |  |
|  | Qiang | 10 | 4.13±1.04 |  |  |  |
|  | Yi | 11 | 4.64±0.89 |  |  |  |
|  | Hui | 8 | 5.04±0.86 |  |  |  |
| Nurse Perceived Organizational Support Scale | Han | 1200 | 3.37±0.76 | 1.879 | 0.112 | / |
|  | Tibetan | 34 | 3.04±0.94 |  |  |  |
|  | Qiang | 10 | 3.34±0.85 |  |  |  |
|  | Yi | 11 | 3.47±0.38 |  |  |  |
|  | Hui | 8 | 3.66±0.42 |  |  |  |
| emotional support | Han | 1200 | 3.3±0.83 | 2.178 | 0.069 | / |
|  | Tibetan | 34 | 2.94±0.99 |  |  |  |
|  | Qiang | 10 | 3.34±0.9 |  |  |  |
|  | Yi | 11 | 3.45±0.39 |  |  |  |
|  | Hui | 8 | 3.7±0.46 |  |  |  |
| instrumental support | Han | 1200 | 3.58±0.81 | 0.794 | 0.529 | / |
|  | Tibetan | 34 | 3.37±1.01 |  |  |  |
|  | Qiang | 10 | 3.33±0.99 |  |  |  |
|  | Yi | 11 | 3.52±0.69 |  |  |  |
|  | Hui | 8 | 3.54±0.83 |  |  |  |
| Note：1 means Han, 2 means Tibetan, 3 means Qiang, 4 means Yi, 5 means Hui. | | | | | | |

## The difference test of the variables in different education level (N=1263)

| Scale and its dimensions | group | n | score _mean_（$\bar{x}\pm s$） | *F* value | *P* value | multiple comparisons |
| --- | --- | --- | --- | --- | --- | --- |
| Chinese Nurse Job Stressors Scale | technical secondary school | 12 | 2.75±0.40 | 1.754 | 0.154 | / |
|  | junior college | 248 | 2.63±0.51 |  |  |  |
|  | bachelor's degree | 988 | 2.57±0.57 |  |  |  |
|  | master's degree | 15 | 2.75±0.55 |  |  |  |
| nursing profession and career issues | technical secondary school | 12 | 2.95±0.68 | 1.607 | 0.186 | / |
|  | junior college | 248 | 2.80±0.63 |  |  |  |
|  | bachelor's degree | 988 | 2.72±0.69 |  |  |  |
|  | master's degree | 15 | 2.92±0.69 |  |  |  |
| workload and time pressure | technical secondary school | 12 | 3.02±0.45 | 1.236 | 0.295 | / |
|  | junior college | 248 | 2.95±0.67 |  |  |  |
|  | bachelor's degree | 988 | 2.88±0.71 |  |  |  |
|  | master's degree | 15 | 3.12±0.64 |  |  |  |
| resource and environmental problems | technical secondary school | 12 | 2.47±0.76 | 2.357 | 0.07 | / |
|  | junior college | 248 | 2.49±0.71 |  |  |  |
|  | bachelor's degree | 988 | 2.37±0.73 |  |  |  |
|  | master's degree | 15 | 2.67±0.58 |  |  |  |
| patient care and interaction | technical secondary school | 12 | 2.63±0.68 | 0.675 | 0.567 | / |
|  | junior college | 248 | 2.55±0.58 |  |  |  |
|  | bachelor's degree | 988 | 2.51±0.65 |  |  |  |
|  | master's degree | 15 | 2.65±0.68 |  |  |  |
| interpersonal relationships and manage ment issues | technical secondary school | 12 | 2.69±0.46 | 1.493 | 0.215 | / |
|  | junior college | 248 | 2.48±0.66 |  |  |  |
|  | bachelor's degree | 988 | 2.41±0.68 |  |  |  |
|  | master's degree | 15 | 2.55±0.75 |  |  |  |
| Decisional Fatigue Scale | technical secondary school | 12 | 2.45±0.60 | 0.851 | 0.466 | / |
|  | junior college | 248 | 2.33±0.65 |  |  |  |
|  | bachelor's degree | 988 | 2.27±0.66 |  |  |  |
|  | master's degree | 15 | 2.40±0.61 |  |  |  |
| Nurse Psychological Capital Questionnaire | technical secondary school | 12 | 4.45±0.60 | 1.396 | 0.242 | / |
|  | junior college | 248 | 4.38±0.71 |  |  |  |
|  | bachelor's degree | 988 | 4.46±0.70 |  |  |  |
|  | master's degree | 15 | 4.22±0.69 |  |  |  |
| self-efficacy | technical secondary school | 12 | 4.65±0.51 | 0.756 | 0.519 | / |
|  | junior college | 248 | 4.48±0.82 |  |  |  |
|  | bachelor's degree | 988 | 4.55±0.80 |  |  |  |
|  | master's degree | 15 | 4.43±0.66 |  |  |  |
| hope | technical secondary school | 12 | 4.28±0.84 | 0.905 | 0.438 | / |
|  | junior college | 248 | 4.36±0.86 |  |  |  |
|  | bachelor's degree | 988 | 4.38±0.86 |  |  |  |
|  | master's degree | 15 | 4.02±0.75 |  |  |  |
| resilience | technical secondary school | 12 | 4.52±0.72 | 2.028 | 0.108 | / |
|  | junior college | 248 | 4.35±0.85 |  |  |  |
|  | bachelor's degree | 988 | 4.48±0.83 |  |  |  |
|  | master's degree | 15 | 4.21±0.74 |  |  |  |
| optimism | technical secondary school | 12 | 4.31±0.83 | 1.734 | 0.158 | / |
|  | junior college | 248 | 4.27±0.91 |  |  |  |
|  | bachelor's degree | 988 | 4.41±0.91 |  |  |  |
|  | master's degree | 15 | 4.22±1.04 |  |  |  |
| Nurse Perceived Organizational Support Scale | technical secondary school | 12 | 2.92±0.57 | 18.476 | 0 | 1＜3；1＜4；2＜3；2＜4 |
|  | junior college | 248 | 3.06±0.73 |  |  |  |
|  | bachelor's degree | 988 | 3.44±0.76 |  |  |  |
|  | master's degree | 15 | 3.65±0.78 |  |  |  |
| emotional support | technical secondary school | 12 | 2.87±0.63 | 19.109 | 0 | 1＜3；1＜4；2＜3；2＜4 |
|  | junior college | 248 | 2.97±0.78 |  |  |  |
|  | bachelor's degree | 988 | 3.38±0.82 |  |  |  |
|  | master's degree | 15 | 3.61±0.83 |  |  |  |
| instrumental support | technical secondary school | 12 | 3.08±0.65 | 7.623 | 0 | 1＜3；1＜4；2＜3 |
|  | junior college | 248 | 3.38±0.77 |  |  |  |
|  | bachelor's degree | 988 | 3.63±0.82 |  |  |  |
|  | master's degree | 15 | 3.76±0.88 |  |  |  |
| Note：1 means technical secondary school, 2 means junior college, 3 means bachelor's degree, 4 means master's degree. | | | | | | |

## The difference test of the variables in different hospital type (N=1263)

| Scale and its dimensions | group | n | score _mean_（$\bar{x}\pm s$） | *F* value | *P* value |
| --- | --- | --- | --- | --- | --- |
| Chinese Nurse Job Stressors Scale | general hospital | 924 | 2.59±0.57 | 0.982 | 0.327 |
|  | specialty hospital | 339 | 2.56±0.52 |  |  |
| nursing profession and career issues | general hospital | 924 | 2.75±0.68 | 0.933 | 0.351 |
|  | specialty hospital | 339 | 2.71±0.65 |  |  |
| workload and time pressure | general hospital | 924 | 2.91±0.71 | 1.353 | 0.176 |
|  | specialty hospital | 339 | 2.85±0.67 |  |  |
| resource and environmental problems | general hospital | 924 | 2.4±0.74 | 0.195 | 0.846 |
|  | specialty hospital | 339 | 2.39±0.68 |  |  |
| patient care and interaction | general hospital | 924 | 2.52±0.64 | 0.514 | 0.607 |
|  | specialty hospital | 339 | 2.50±0.63 |  |  |
| interpersonal relationships and manage ment issues | general hospital | 924 | 2.44±0.69 | 0.864 | 0.388 |
|  | specialty hospital | 339 | 2.40±0.65 |  |  |
| Decisional Fatigue Scale | general hospital | 924 | 2.31±0.67 | 2.235 | 0.026 |
|  | specialty hospital | 339 | 2.22±0.63 |  |  |
| Nurse Psychological Capital Questionnaire | general hospital | 924 | 4.43±0.72 | -1.222 | 0.222 |
|  | specialty hospital | 339 | 4.48±0.65 |  |  |
| self-efficacy | general hospital | 924 | 4.55±0.82 | 0.630 | 0.528 |
|  | specialty hospital | 339 | 4.51±0.77 |  |  |
| hope | general hospital | 924 | 4.34±0.88 | -1.903 | 0.057 |
|  | specialty hospital | 339 | 4.44±0.81 |  |  |
| resilience | general hospital | 924 | 4.43±0.84 | -1.601 | 0.110 |
|  | specialty hospital | 339 | 4.51±0.80 |  |  |
| optimism | general hospital | 924 | 4.37±0.93 | -1.107 | 0.269 |
|  | specialty hospital | 339 | 4.43±0.86 |  |  |
| Nurse Perceived Organizational Support Scale | general hospital | 924 | 3.32±0.79 | -3.550 | 0.000 |
|  | specialty hospital | 339 | 3.48±0.70 |  |  |
| emotional support | general hospital | 924 | 3.25±0.85 | -3.300 | 0.001 |
|  | specialty hospital | 339 | 3.42±0.76 |  |  |
| instrumental support | general hospital | 924 | 3.53±0.84 | -3.178 | 0.002 |
|  | specialty hospital | 339 | 3.69±0.75 |  |  |

## The difference test of the variables in different hospital nature (N=1263)

| Scale and its dimensions | group | n | score _mean_（$\bar{x}\pm s$） | *F* value | *P* value |
| --- | --- | --- | --- | --- | --- |
| Chinese Nurse Job Stressors Scale | public hospital | 1005 | 2.52±0.57 | -9.132 | 0.000 |
|  | private hospital | 258 | 2.83±0.45 |  |  |
| nursing profession and career issues | public hospital | 1005 | 2.68±0.68 | -7.102 | 0.000 |
|  | private hospital | 258 | 2.98±0.58 |  |  |
| workload and time pressure | public hospital | 1005 | 2.84±0.71 | -6.802 | 0.000 |
|  | private hospital | 258 | 3.13±0.60 |  |  |
| resource and environmental problems | public hospital | 1005 | 2.35±0.73 | -4.742 | 0.000 |
|  | private hospital | 258 | 2.59±0.66 |  |  |
| patient care and interaction | public hospital | 1005 | 2.46±0.64 | -7.803 | 0.000 |
|  | private hospital | 258 | 2.77±0.56 |  |  |
| interpersonal relationships and manage ment issues | public hospital | 1005 | 2.36±0.69 | -7.675 | 0.000 |
|  | private hospital | 258 | 2.68±0.58 |  |  |
| Decisional Fatigue Scale | public hospital | 1005 | 2.25±0.65 | -4.118 | 0.000 |
|  | private hospital | 258 | 2.44±0.67 |  |  |
| Nurse Psychological Capital Questionnaire | public hospital | 1005 | 4.5±0.7 | 5.569 | 0.000 |
|  | private hospital | 258 | 4.23±0.66 |  |  |
| self-efficacy | public hospital | 1005 | 4.59±0.8 | 4.219 | 0.000 |
|  | private hospital | 258 | 4.35±0.77 |  |  |
| hope | public hospital | 1005 | 4.43±0.86 | 4.777 | 0.000 |
|  | private hospital | 258 | 4.14±0.84 |  |  |
| resilience | public hospital | 1005 | 4.5±0.84 | 4.659 | 0.000 |
|  | private hospital | 258 | 4.23±0.8 |  |  |
| optimism | public hospital | 1005 | 4.45±0.9 | 4.983 | 0.000 |
|  | private hospital | 258 | 4.13±0.9 |  |  |
| Nurse Perceived Organizational Support Scale | public hospital | 1005 | 3.39±0.76 | 2.592 | 0.010 |
|  | private hospital | 258 | 3.25±0.78 |  |  |
| emotional support | public hospital | 1005 | 3.33±0.82 | 2.707 | 0.007 |
|  | private hospital | 258 | 3.17±0.84 |  |  |
| instrumental support | public hospital | 1005 | 3.59±0.82 | 1.41 | 0.159 |
|  | private hospital | 258 | 3.51±0.82 |  |  |

## The difference test of the variables in different hospital grade (N=1263)

| Scale and its dimensions | group | n | score _mean_（$\bar{x}\pm s$） | *F* value | *P* value | multiple comparisons |
| --- | --- | --- | --- | --- | --- | --- |
| Chinese Nurse Job Stressors Scale | primary A level | 41 | 2.79±0.54 | 2.758 | 0.017 | 1＞3；1＞4；1＞6；  5＞3；5＞6 |
|  | Primary B level | 5 | 2.47±0.34 |  |  |  |
|  | secondary A level | 292 | 2.54±0.37 |  |  |  |
|  | secondary B level | 134 | 2.54±0.39 |  |  |  |
|  | tertiary A level | 487 | 2.63±0.69 |  |  |  |
|  | tertiary B level | 304 | 2.55±0.53 |  |  |  |
| nursing profession and career issues | primary A level | 41 | 2.94±0.67 | 2.231 | 0.049 | 1＞2；1＞3；5＞3 |
|  | Primary B level | 5 | 2.26±0.4 |  |  |  |
|  | secondary A level | 292 | 2.68±0.56 |  |  |  |
|  | secondary B level | 134 | 2.7±0.55 |  |  |  |
|  | tertiary A level | 487 | 2.79±0.78 |  |  |  |
|  | tertiary B level | 304 | 2.73±0.65 |  |  |  |
| workload and time pressure | primary A level | 41 | 3.1±0.64 | 1.153 | 0.331 | / |
|  | Primary B level | 5 | 2.64±0.36 |  |  |  |
|  | secondary A level | 292 | 2.89±0.59 |  |  |  |
|  | secondary B level | 134 | 2.89±0.57 |  |  |  |
|  | tertiary A level | 487 | 2.92±0.79 |  |  |  |
|  | tertiary B level | 304 | 2.85±0.69 |  |  |  |
| resource and environmental problems | primary A level | 41 | 2.55±0.77 | 0.835 | 0.525 | / |
|  | Primary B level | 5 | 2.27±0.72 |  |  |  |
|  | secondary A level | 292 | 2.36±0.62 |  |  |  |
|  | secondary B level | 134 | 2.35±0.62 |  |  |  |
|  | tertiary A level | 487 | 2.42±0.82 |  |  |  |
|  | tertiary B level | 304 | 2.42±0.69 |  |  |  |
| patient care and interaction | primary A level | 41 | 2.72±0.61 | 3.302 | 0.006 | 1＞3；1＞4；1＞6；5＞3；5＞4；5＞6 |
|  | Primary B level | 5 | 2.6±0.55 |  |  |  |
|  | secondary A level | 292 | 2.46±0.5 |  |  |  |
|  | secondary B level | 134 | 2.43±0.49 |  |  |  |
|  | tertiary A level | 487 | 2.59±0.75 |  |  |  |
|  | tertiary B level | 304 | 2.48±0.6 |  |  |  |
| interpersonal relationships and manage ment issues | primary A level | 41 | 2.68±0.63 | 2.356 | 0.039 | 1＞3；1＞4；1＞6；5＞6 |
|  | Primary B level | 5 | 2.47±0.44 |  |  |  |
|  | secondary A level | 292 | 2.38±0.54 |  |  |  |
|  | secondary B level | 134 | 2.41±0.59 |  |  |  |
|  | tertiary A level | 487 | 2.48±0.78 |  |  |  |
|  | tertiary B level | 304 | 2.37±0.66 |  |  |  |
| Decisional Fatigue Scale | primary A level | 41 | 2.41±0.65 | 1.612 | 0.154 | / |
|  | Primary B level | 5 | 2.18±0.58 |  |  |  |
|  | secondary A level | 292 | 2.2±0.56 |  |  |  |
|  | secondary B level | 134 | 2.31±0.55 |  |  |  |
|  | tertiary A level | 487 | 2.32±0.73 |  |  |  |
|  | tertiary B level | 304 | 2.29±0.66 |  |  |  |
| Nurse Psychological Capital Questionnaire | primary A level | 41 | 4.32±0.75 | 0.95 | 0.447 | / |
|  | Primary B level | 5 | 4.57±0.63 |  |  |  |
|  | secondary A level | 292 | 4.48±0.53 |  |  |  |
|  | secondary B level | 134 | 4.51±0.6 |  |  |  |
|  | tertiary A level | 487 | 4.41±0.81 |  |  |  |
|  | tertiary B level | 304 | 4.43±0.7 |  |  |  |
| self-efficacy | primary A level | 41 | 4.38±0.9 | 1.359 | 0.237 | / |
|  | Primary B level | 5 | 4.77±0.79 |  |  |  |
|  | secondary A level | 292 | 4.61±0.68 |  |  |  |
|  | secondary B level | 134 | 4.6±0.71 |  |  |  |
|  | tertiary A level | 487 | 4.49±0.89 |  |  |  |
|  | tertiary B level | 304 | 4.53±0.8 |  |  |  |
| hope | primary A level | 41 | 4.21±0.84 | 0.678 | 0.64 | / |
|  | Primary B level | 5 | 4.3±0.86 |  |  |  |
|  | secondary A level | 292 | 4.43±0.71 |  |  |  |
|  | secondary B level | 134 | 4.4±0.84 |  |  |  |
|  | tertiary A level | 487 | 4.34±0.93 |  |  |  |
|  | tertiary B level | 304 | 4.36±0.89 |  |  |  |
| resilience | primary A level | 41 | 4.36±0.93 | 0.651 | 0.661 | / |
|  | Primary B level | 5 | 4.8±0.51 |  |  |  |
|  | secondary A level | 292 | 4.45±0.7 |  |  |  |
|  | secondary B level | 134 | 4.54±0.76 |  |  |  |
|  | tertiary A level | 487 | 4.44±0.93 |  |  |  |
|  | tertiary B level | 304 | 4.43±0.82 |  |  |  |
| optimism | primary A level | 41 | 4.34±0.99 | 0.673 | 0.644 | / |
|  | Primary B level | 5 | 4.33±0.85 |  |  |  |
|  | secondary A level | 292 | 4.41±0.82 |  |  |  |
|  | secondary B level | 134 | 4.5±0.86 |  |  |  |
|  | tertiary A level | 487 | 4.35±0.96 |  |  |  |
|  | tertiary B level | 304 | 4.36±0.92 |  |  |  |
| Nurse Perceived Organizational Support Scale | primary A level | 41 | 2.85±0.65 | 5.423 | 0.000 | 3＞1；4＞1；5＞1；6＞1；6＞4 |
|  | Primary B level | 5 | 2.86±0.26 |  |  |  |
|  | secondary A level | 292 | 3.36±0.75 |  |  |  |
|  | secondary B level | 134 | 3.26±0.78 |  |  |  |
|  | tertiary A level | 487 | 3.39±0.81 |  |  |  |
|  | tertiary B level | 304 | 3.44±0.69 |  |  |  |
| emotional support | primary A level | 41 | 2.77±0.72 | 5.312 | 0.000 | 3＞1；4＞1；5＞1；6＞1；6＞4 |
|  | Primary B level | 5 | 2.66±0.35 |  |  |  |
|  | secondary A level | 292 | 3.3±0.81 |  |  |  |
|  | secondary B level | 134 | 3.18±0.85 |  |  |  |
|  | tertiary A level | 487 | 3.33±0.88 |  |  |  |
|  | tertiary B level | 304 | 3.38±0.74 |  |  |  |
| instrumental support | primary A level | 41 | 3.12±0.68 | 3.13 | 0.008 | 3＞1；4＞1；5＞1；6＞1 |
|  | Primary B level | 5 | 3.53±0.38 |  |  |  |
|  | secondary A level | 292 | 3.58±0.77 |  |  |  |
|  | secondary B level | 134 | 3.51±0.83 |  |  |  |
|  | tertiary A level | 487 | 3.59±0.87 |  |  |  |
|  | tertiary B level | 304 | 3.64±0.78 |  |  |  |
| Note：1 means primary A level, 2 means Primary B level, 3 means secondary A level, 4 means secondary B level, 5 means tertiary A level, 6 menas tertiary B level. | | | | | | |

## The difference test of the variables in different employment type (N=1263)

| Scale and its dimensions | group | n | score _mean_（$\bar{x}\pm s$） | *F* value | *P* value | multiple comparisons |
| --- | --- | --- | --- | --- | --- | --- |
| Chinese Nurse Job Stressors Scale | bianzhi | 240 | 2.66±0.52 | 3.409 | 0.033 | 1＞2 |
|  | contract-based | 985 | 2.57±0.56 |  |  |  |
|  | personnel agent | 38 | 2.48±0.63 |  |  |  |
| nursing profession and career issues | bianzhi | 240 | 2.83±0.66 | 2.978 | 0.051 | / |
|  | contract-based | 985 | 2.73±0.67 |  |  |  |
|  | personnel agent | 38 | 2.61±0.77 |  |  |  |
| workload and time pressure | bianzhi | 240 | 3.01±0.66 | 3.797 | 0.023 | 1＞2 |
|  | contract-based | 985 | 2.87±0.7 |  |  |  |
|  | personnel agent | 38 | 2.82±0.76 |  |  |  |
| resource and environmental problems | bianzhi | 240 | 2.47±0.7 | 2.247 | 0.106 | / |
|  | contract-based | 985 | 2.39±0.73 |  |  |  |
|  | personnel agent | 38 | 2.23±0.67 |  |  |  |
| patient care and interaction | bianzhi | 240 | 2.59±0.6 | 1.737 | 0.176 | / |
|  | contract-based | 985 | 2.5±0.64 |  |  |  |
|  | personnel agent | 38 | 2.52±0.66 |  |  |  |
| interpersonal relationships and manage ment issues | bianzhi | 240 | 2.5±0.66 | 3.024 | 0.049 | 1＞3 |
|  | contract-based | 985 | 2.42±0.68 |  |  |  |
|  | personnel agent | 38 | 2.23±0.74 |  |  |  |
| Decisional Fatigue Scale | bianzhi | 240 | 2.32±0.64 | 0.438 | 0.646 | / |
|  | contract-based | 985 | 2.28±0.66 |  |  |  |
|  | personnel agent | 38 | 2.32±0.7 |  |  |  |
| Nurse Psychological Capital Questionnaire | bianzhi | 240 | 4.36±0.67 | 2.092 | 0.124 | / |
|  | contract-based | 985 | 4.46±0.71 |  |  |  |
|  | personnel agent | 38 | 4.54±0.74 |  |  |  |
| self-efficacy | bianzhi | 240 | 4.49±0.75 | 1.145 | 0.318 | / |
|  | contract-based | 985 | 4.54±0.81 |  |  |  |
|  | personnel agent | 38 | 4.7±0.9 |  |  |  |
| hope | bianzhi | 240 | 4.27±0.85 | 2.101 | 0.123 | / |
|  | contract-based | 985 | 4.39±0.87 |  |  |  |
|  | personnel agent | 38 | 4.41±0.85 |  |  |  |
| resilience | bianzhi | 240 | 4.34±0.82 | 2.389 | 0.092 | / |
|  | contract-based | 985 | 4.47±0.84 |  |  |  |
|  | personnel agent | 38 | 4.51±0.81 |  |  |  |
| optimism | bianzhi | 240 | 4.33±0.86 | 0.979 | 0.376 | / |
|  | contract-based | 985 | 4.39±0.92 |  |  |  |
|  | personnel agent | 38 | 4.54±1.02 |  |  |  |
| Nurse Perceived Organizational Support Scale | bianzhi | 240 | 3.48±0.82 | 4.993 | 0.007 | 1＞2 |
|  | contract-based | 985 | 3.33±0.74 |  |  |  |
|  | personnel agent | 38 | 3.54±1.08 |  |  |  |
| emotional support | bianzhi | 240 | 3.42±0.89 | 4.527 | 0.011 | 1＞2 |
|  | contract-based | 985 | 3.26±0.79 |  |  |  |
|  | personnel agent | 38 | 3.47±1.19 |  |  |  |
| instrumental support | bianzhi | 240 | 3.68±0.83 | 3.694 | 0.025 | 1＞2 |
|  | contract-based | 985 | 3.54±0.81 |  |  |  |
|  | personnel agent | 38 | 3.77±0.91 |  |  |  |
| Note：1 means bianzhi, 2 means contract-based, 3 means personnel agent. | | | | | | |

## The difference test of the variables in different work experience (N=1263)

| Scale and its dimensions | group | n | score _mean_（$\bar{x}\pm s$） | *F* value | *P* value | multiple comparisons |
| --- | --- | --- | --- | --- | --- | --- |
| Chinese Nurse Job Stressors Scale | ＜5 years | 214 | 2.66±0.56 | 3.461 | 0.008 | 1＞3；1＞4；1＞5；  2＞4；2＞5 |
|  | 5-10 years | 436 | 2.62±0.52 |  |  |  |
|  | 11-20 years | 439 | 2.56±0.56 |  |  |  |
|  | 21-30 years | 136 | 2.50±0.60 |  |  |  |
|  | ＞30 years | 38 | 2.40±0.68 |  |  |  |
| nursing profession and career issues | ＜5 years | 214 | 2.84±0.63 | 3.216 | 0.012 | 1＞3；1＞4；1＞5；2＞4；2＞5 |
|  | 5-10 years | 436 | 2.78±0.67 |  |  |  |
|  | 11-20 years | 439 | 2.72±0.67 |  |  |  |
|  | 21-30 years | 136 | 2.64±0.71 |  |  |  |
|  | ＞30 years | 38 | 2.55±0.85 |  |  |  |
| workload and time pressure | ＜5 years | 214 | 2.96±0.67 | 1.503 | 0.199 | // |
|  | 5-10 years | 436 | 2.93±0.65 |  |  |  |
|  | 11-20 years | 439 | 2.86±0.73 |  |  |  |
|  | 21-30 years | 136 | 2.85±0.71 |  |  |  |
|  | ＞30 years | 38 | 2.76±0.89 |  |  |  |
| resource and environmental problems | ＜5 years | 214 | 2.47±0.70 | 2.474 | 0.043 | 1＞5；2＞5；3＞5 |
|  | 5-10 years | 436 | 2.39±0.69 |  |  |  |
|  | 11-20 years | 439 | 2.43±0.75 |  |  |  |
|  | 21-30 years | 136 | 2.32±0.77 |  |  |  |
|  | ＞30 years | 38 | 2.12±0.72 |  |  |  |
| patient care and interaction | ＜5 years | 214 | 2.61±0.65 | 4.504 | 0.001 | 1＞3；1＞4；1＞5；2＞3；2＞4；2＞5 |
|  | 5-10 years | 436 | 2.57±0.60 |  |  |  |
|  | 11-20 years | 439 | 2.48±0.65 |  |  |  |
|  | 21-30 years | 136 | 2.39±0.65 |  |  |  |
|  | ＞30 years | 38 | 2.33±0.71 |  |  |  |
| interpersonal relationships and manage ment issues | ＜5 years | 214 | 2.49±0.71 | 1.283 | 0.275 | / |
|  | 5-10 years | 436 | 2.45±0.66 |  |  |  |
|  | 11-20 years | 439 | 2.4±0.66 |  |  |  |
|  | 21-30 years | 136 | 2.4±0.69 |  |  |  |
|  | ＞30 years | 38 | 2.28±0.83 |  |  |  |
| Decisional Fatigue Scale | ＜5 years | 214 | 2.37±0.65 | 2.006 | 0.091 | / |
|  | 5-10 years | 436 | 2.3±0.65 |  |  |  |
|  | 11-20 years | 439 | 2.27±0.66 |  |  |  |
|  | 21-30 years | 136 | 2.19±0.64 |  |  |  |
|  | ＞30 years | 38 | 2.20±0.70 |  |  |  |
| Nurse Psychological Capital Questionnaire | ＜5 years | 214 | 4.33±0.75 | 2.386 | 0.049 | 2＞1；4＞1；5＞1 |
|  | 5-10 years | 436 | 4.47±0.68 |  |  |  |
|  | 11-20 years | 439 | 4.44±0.70 |  |  |  |
|  | 21-30 years | 136 | 4.5±0.68 |  |  |  |
|  | ＞30 years | 38 | 4.61±0.76 |  |  |  |
| self-efficacy | ＜5 years | 214 | 4.42±0.87 | 2.125 | 0.076 | / |
|  | 5-10 years | 436 | 4.56±0.79 |  |  |  |
|  | 11-20 years | 439 | 4.54±0.80 |  |  |  |
|  | 21-30 years | 136 | 4.62±0.72 |  |  |  |
|  | ＞30 years | 38 | 4.72±0.83 |  |  |  |
| hope | ＜5 years | 214 | 4.26±0.90 | 1.486 | 0.204 | / |
|  | 5-10 years | 436 | 4.38±0.84 |  |  |  |
|  | 11-20 years | 439 | 4.37±0.86 |  |  |  |
|  | 21-30 years | 136 | 4.45±0.83 |  |  |  |
|  | ＞30 years | 38 | 4.54±0.91 |  |  |  |
| resilience | ＜5 years | 214 | 4.34±0.87 | 1.639 | 0.162 | / |
|  | 5-10 years | 436 | 4.50±0.80 |  |  |  |
|  | 11-20 years | 439 | 4.45±0.85 |  |  |  |
|  | 21-30 years | 136 | 4.41±0.84 |  |  |  |
|  | ＞30 years | 38 | 4.62±0.84 |  |  |  |
| optimism | ＜5 years | 214 | 4.23±0.94 | 2.349 | 0.053 | / |
|  | 5-10 years | 436 | 4.41±0.91 |  |  |  |
|  | 11-20 years | 439 | 4.38±0.91 |  |  |  |
|  | 21-30 years | 136 | 4.50±0.87 |  |  |  |
|  | ＞30 years | 38 | 4.53±0.85 |  |  |  |
| Nurse Perceived Organizational Support Scale | ＜5 years | 214 | 3.00±0.73 | 15.69 | 0.000 | 2＞1；3＞1；4＞1；5＞1 |
|  | 5-10 years | 436 | 3.40±0.78 |  |  |  |
|  | 11-20 years | 439 | 3.45±0.76 |  |  |  |
|  | 21-30 years | 136 | 3.50±0.68 |  |  |  |
|  | ＞30 years | 38 | 3.48±0.65 |  |  |  |
| emotional support | ＜5 years | 214 | 2.90±0.78 | 16.01 | 0.000 | 2＞1；3＞1；4＞1；5＞1 |
|  | 5-10 years | 436 | 3.34±0.84 |  |  |  |
|  | 11-20 years | 439 | 3.39±0.82 |  |  |  |
|  | 21-30 years | 136 | 3.45±0.73 |  |  |  |
|  | ＞30 years | 38 | 3.45±0.72 |  |  |  |
| instrumental support | ＜5 years | 214 | 3.31±0.80 | 6.899 | 0.000 | 2＞1；3＞1；4＞1 |
|  | 5-10 years | 436 | 3.61±0.81 |  |  |  |
|  | 11-20 years | 439 | 3.64±0.84 |  |  |  |
|  | 21-30 years | 136 | 3.66±0.75 |  |  |  |
|  | ＞30 years | 38 | 3.55±0.67 |  |  |  |
| Note：1 means ≤＜5 years, 2 means 5-10 years, 3 means 11-20 years, 4 means 21-30 years, 5 means ＞30 years. | | | | | | |

## The difference test of the variables in different professional title (N=1263)

| Scale and its dimensions | group | n | score _mean_（$\bar{x}\pm s$） | *F* value | *P* value | multiple comparisons |
| --- | --- | --- | --- | --- | --- | --- |
| Chinese Nurse Job Stressors Scale | staff nurse | 183 | 2.62±0.51 | 0.493 | 0.741 | / |
|  | primary nurse | 387 | 2.59±0.55 |  |  |  |
|  | nurse-in-charge | 594 | 2.57±0.59 |  |  |  |
|  | associate chief nurse | 93 | 2.57±0.51 |  |  |  |
|  | chief nurse | 6 | 2.81±0.34 |  |  |  |
| nursing profession and career issues | staff nurse | 183 | 2.8±0.62 | 0.467 | 0.76 | / |
|  | primary nurse | 387 | 2.74±0.66 |  |  |  |
|  | nurse-in-charge | 594 | 2.73±0.71 |  |  |  |
|  | associate chief nurse | 93 | 2.73±0.64 |  |  |  |
|  | chief nurse | 6 | 2.81±0.55 |  |  |  |
| workload and time pressure | staff nurse | 183 | 2.93±0.64 | 0.115 | 0.977 | / |
|  | primary nurse | 387 | 2.89±0.69 |  |  |  |
|  | nurse-in-charge | 594 | 2.89±0.72 |  |  |  |
|  | associate chief nurse | 93 | 2.9±0.66 |  |  |  |
|  | chief nurse | 6 | 2.90±0.80 |  |  |  |
| resource and environmental problems | staff nurse | 183 | 2.40±0.69 | 0.28 | 0.891 | / |
|  | primary nurse | 387 | 2.42±0.71 |  |  |  |
|  | nurse-in-charge | 594 | 2.40±0.76 |  |  |  |
|  | associate chief nurse | 93 | 2.35±0.67 |  |  |  |
|  | chief nurse | 6 | 2.61±0.25 |  |  |  |
| patient care and interaction | staff nurse | 183 | 2.54±0.60 | 0.787 | 0.533 | / |
|  | primary nurse | 387 | 2.54±0.63 |  |  |  |
|  | nurse-in-charge | 594 | 2.50±0.66 |  |  |  |
|  | associate chief nurse | 93 | 2.47±0.61 |  |  |  |
|  | chief nurse | 6 | 2.85±0.30 |  |  |  |
| interpersonal relationships and manage ment issues | staff nurse | 183 | 2.46±0.63 | 0.647 | 0.629 | / |
|  | primary nurse | 387 | 2.42±0.68 |  |  |  |
|  | nurse-in-charge | 594 | 2.41±0.70 |  |  |  |
|  | associate chief nurse | 93 | 2.46±0.64 |  |  |  |
|  | chief nurse | 6 | 2.78±0.47 |  |  |  |
| Decisional Fatigue Scale | staff nurse | 183 | 2.31±0.62 | 0.138 | 0.968 | / |
|  | primary nurse | 387 | 2.28±0.65 |  |  |  |
|  | nurse-in-charge | 594 | 2.28±0.69 |  |  |  |
|  | associate chief nurse | 93 | 2.31±0.59 |  |  |  |
|  | chief nurse | 6 | 2.17±0.55 |  |  |  |
| Nurse Psychological Capital Questionnaire | staff nurse | 183 | 4.39±0.70 | 0.814 | 0.516 | / |
|  | primary nurse | 387 | 4.47±0.72 |  |  |  |
|  | nurse-in-charge | 594 | 4.45±0.70 |  |  |  |
|  | associate chief nurse | 93 | 4.37±0.65 |  |  |  |
|  | chief nurse | 6 | 4.22±0.42 |  |  |  |
| self-efficacy | staff nurse | 183 | 4.50±0.85 | 0.808 | 0.52 | / |
|  | primary nurse | 387 | 4.58±0.82 |  |  |  |
|  | nurse-in-charge | 594 | 4.54±0.79 |  |  |  |
|  | associate chief nurse | 93 | 4.45±0.75 |  |  |  |
|  | chief nurse | 6 | 4.28±0.38 |  |  |  |
| hope | staff nurse | 183 | 4.30±0.86 | 0.859 | 0.488 | / |
|  | primary nurse | 387 | 4.38±0.86 |  |  |  |
|  | nurse-in-charge | 594 | 4.40±0.87 |  |  |  |
|  | associate chief nurse | 93 | 4.27±0.85 |  |  |  |
|  | chief nurse | 6 | 4.22±0.87 |  |  |  |
| resilience | staff nurse | 183 | 4.42±0.79 | 0.553 | 0.697 | / |
|  | primary nurse | 387 | 4.46±0.83 |  |  |  |
|  | nurse-in-charge | 594 | 4.47±0.87 |  |  |  |
|  | associate chief nurse | 93 | 4.37±0.76 |  |  |  |
|  | chief nurse | 6 | 4.13±0.45 |  |  |  |
| optimism | staff nurse | 183 | 4.28±0.93 | 0.753 | 0.556 | / |
|  | primary nurse | 387 | 4.41±0.93 |  |  |  |
|  | nurse-in-charge | 594 | 4.39±0.92 |  |  |  |
|  | associate chief nurse | 93 | 4.42±0.77 |  |  |  |
|  | chief nurse | 6 | 4.22±0.81 |  |  |  |
| Nurse Perceived Organizational Support Scale | staff nurse | 183 | 2.92±0.66 | 21.925 | 0.000 | 2＞1；3＞1；4＞1；5＞1；3＞2；4＞2 |
|  | primary nurse | 387 | 3.33±0.75 |  |  |  |
|  | nurse-in-charge | 594 | 3.48±0.77 |  |  |  |
|  | associate chief nurse | 93 | 3.55±0.66 |  |  |  |
|  | chief nurse | 6 | 3.73±0.21 |  |  |  |
| emotional support | staff nurse | 183 | 2.83±0.72 | 21.312 | 0.000 | 2＞1；3＞1；4＞1；5＞1；3＞2；4＞2 |
|  | primary nurse | 387 | 3.26±0.82 |  |  |  |
|  | nurse-in-charge | 594 | 3.43±0.83 |  |  |  |
|  | associate chief nurse | 93 | 3.51±0.73 |  |  |  |
|  | chief nurse | 6 | 3.7±0.11 |  |  |  |
| instrumental support | staff nurse | 183 | 3.23±0.75 | 11.644 | 0.000 | 2＞1；3＞1；4＞1；3＞2 |
|  | primary nurse | 387 | 3.56±0.81 |  |  |  |
|  | nurse-in-charge | 594 | 3.68±0.84 |  |  |  |
|  | associate chief nurse | 93 | 3.69±0.67 |  |  |  |
|  | chief nurse | 6 | 3.83±0.59 |  |  |  |
| Note：1 means staff nurse, 2 means primary nurse, 3 means nurse-in-charge, 4 means associate chief nurse, 5 means chief nurse. | | | | | | |

## The difference test of the variables in different professional position (N=1263)

| Scale and its dimensions | group | n | score _mean_（$\bar{x}\pm s$） | *F* value | *P* value | multiple comparisons |
| --- | --- | --- | --- | --- | --- | --- |
| Chinese Nurse Job Stressors Scale | none | 1140 | 2.58±0.56 | 1.417 | 0.243 | / |
|  | deputy midwife (deputy head nurse) | 58 | 2.65±0.56 |  |  |  |
|  | midwives (head nurses) and above | 65 | 2.67±0.46 |  |  |  |
| nursing profession and career issues | none | 1140 | 2.74±0.68 | 1.069 | 0.344 | / |
|  | deputy midwife (deputy head nurse) | 58 | 2.87±0.60 |  |  |  |
|  | midwives (head nurses) and above | 65 | 2.76±0.64 |  |  |  |
| workload and time pressure | none | 1140 | 2.89±0.7 | 0.712 | 0.491 | / |
|  | deputy midwife (deputy head nurse) | 58 | 2.95±0.74 |  |  |  |
|  | midwives (head nurses) and above | 65 | 2.98±0.62 |  |  |  |
| resource and environmental problems | none | 1140 | 2.39±0.73 | 0.390 | 0.677 | / |
|  | deputy midwife (deputy head nurse) | 58 | 2.45±0.71 |  |  |  |
|  | midwives (head nurses) and above | 65 | 2.46±0.66 |  |  |  |
| patient care and interaction | none | 1140 | 2.51±0.64 | 0.949 | 0.387 | / |
|  | deputy midwife (deputy head nurse) | 58 | 2.54±0.60 |  |  |  |
|  | midwives (head nurses) and above | 65 | 2.62±0.56 |  |  |  |
| interpersonal relationships and manage ment issues | none | 1140 | 2.41±0.68 | 2.255 | 0.105 | / |
|  | deputy midwife (deputy head nurse) | 58 | 2.53±0.68 |  |  |  |
|  | midwives (head nurses) and above | 65 | 2.57±0.54 |  |  |  |
| Decisional Fatigue Scale | none | 1140 | 2.27±0.66 | 3.065 | 0.047 | 2＞1 |
|  | deputy midwife (deputy head nurse) | 58 | 2.45±0.66 |  |  |  |
|  | midwives (head nurses) and above | 65 | 2.40±0.60 |  |  |  |
| Nurse Psychological Capital Questionnaire | none | 1140 | 4.44±0.71 | 0.300 | 0.741 | / |
|  | deputy midwife (deputy head nurse) | 58 | 4.46±0.62 |  |  |  |
|  | midwives (head nurses) and above | 65 | 4.38±0.58 |  |  |  |
| self-efficacy | none | 1140 | 4.54±0.82 | 0.151 | 0.860 | / |
|  | deputy midwife (deputy head nurse) | 58 | 4.59±0.68 |  |  |  |
|  | midwives (head nurses) and above | 65 | 4.53±0.66 |  |  |  |
| hope | none | 1140 | 4.37±0.87 | 0.887 | 0.412 | / |
|  | deputy midwife (deputy head nurse) | 58 | 4.42±0.81 |  |  |  |
|  | midwives (head nurses) and above | 65 | 4.24±0.78 |  |  |  |
| resilience | none | 1140 | 4.46±0.84 | 0.921 | 0.398 | / |
|  | deputy midwife (deputy head nurse) | 58 | 4.40±0.79 |  |  |  |
|  | midwives (head nurses) and above | 65 | 4.32±0.74 |  |  |  |
| optimism | none | 1140 | 4.38±0.93 | 0.157 | 0.854 | / |
|  | deputy midwife (deputy head nurse) | 58 | 4.4±0.82 |  |  |  |
|  | midwives (head nurses) and above | 65 | 4.44±0.71 |  |  |  |
| Nurse Perceived Organizational Support Scale | none | 1140 | 3.36±0.76 | 0.283 | 0.754 | / |
|  | deputy midwife (deputy head nurse) | 58 | 3.43±0.89 |  |  |  |
|  | midwives (head nurses) and above | 65 | 3.33±0.75 |  |  |  |
| emotional support | none | 1140 | 3.3±0.82 | 0.363 | 0.696 | / |
|  | deputy midwife (deputy head nurse) | 58 | 3.38±0.93 |  |  |  |
|  | midwives (head nurses) and above | 65 | 3.26±0.81 |  |  |  |
| instrumental support | none | 1140 | 3.58±0.81 | 0.053 | 0.948 | / |
|  | deputy midwife (deputy head nurse) | 58 | 3.58±1 |  |  |  |
|  | midwives (head nurses) and above | 65 | 3.54±0.78 |  |  |  |
| Note：1 means none, 2 means deputy midwife (deputy head nurse), 3 means midwives (head nurses) and above. | | | | | | |

## The difference test of the variables in different monthly night shifts (N=1263)

| Scale and its dimensions | group | n | score _mean_（$\bar{x}\pm s$） | *F* value | *P* value | multiple comparisons |
| --- | --- | --- | --- | --- | --- | --- |
| Chinese Nurse Job Stressors Scale | 0 | 257 | 2.52±0.56 | 5.707 | 0.001 | 4＞1；4＞2；4＞3 |
|  | 1-4 | 264 | 2.55±0.48 |  |  |  |
|  | 5-9 | 487 | 2.58±0.59 |  |  |  |
|  | ＞9 | 255 | 2.7±0.57 |  |  |  |
| nursing profession and career issues | 0 | 257 | 2.65±0.70 | 3.314 | 0.019 | 4＞1 |
|  | 1-4 | 264 | 2.75±0.61 |  |  |  |
|  | 5-9 | 487 | 2.74±0.70 |  |  |  |
|  | ＞9 | 255 | 2.84±0.67 |  |  |  |
| workload and time pressure | 0 | 257 | 2.83±0.70 | 4.821 | 0.002 | 4＞1；4＞2；4＞3 |
|  | 1-4 | 264 | 2.91±0.65 |  |  |  |
|  | 5-9 | 487 | 2.86±0.72 |  |  |  |
|  | ＞9 | 255 | 3.04±0.69 |  |  |  |
| resource and environmental problems | 0 | 257 | 2.38±0.71 | 4.124 | 0.006 | 4＞1；4＞2；4＞3 |
|  | 1-4 | 264 | 2.34±0.65 |  |  |  |
|  | 5-9 | 487 | 2.37±0.76 |  |  |  |
|  | ＞9 | 255 | 2.54±0.73 |  |  |  |
| patient care and interaction | 0 | 257 | 2.45±0.64 | 6.386 | 0.000 | 4＞1；4＞2；4＞3；2＜3 |
|  | 1-4 | 264 | 2.43±0.56 |  |  |  |
|  | 5-9 | 487 | 2.54±0.66 |  |  |  |
|  | ＞9 | 255 | 2.64±0.64 |  |  |  |
| interpersonal relationships and manage ment issues | 0 | 257 | 2.36±0.66 | 3.573 | 0.014 | 4＞1；4＞2；4＞3 |
|  | 1-4 | 264 | 2.41±0.61 |  |  |  |
|  | 5-9 | 487 | 2.41±0.69 |  |  |  |
|  | ＞9 | 255 | 2.55±0.72 |  |  |  |
| Decisional Fatigue Scale | 0 | 257 | 2.23±0.66 | 1.805 | 0.144 | / |
|  | 1-4 | 264 | 2.31±0.62 |  |  |  |
|  | 5-9 | 487 | 2.27±0.66 |  |  |  |
|  | ＞9 | 255 | 2.35±0.69 |  |  |  |
| Nurse Psychological Capital Questionnaire | 0 | 257 | 4.49±0.71 | 1.032 | 0.377 | / |
|  | 1-4 | 264 | 4.48±0.65 |  |  |  |
|  | 5-9 | 487 | 4.42±0.72 |  |  |  |
|  | ＞9 | 255 | 4.40±0.72 |  |  |  |
| self-efficacy | 0 | 257 | 4.57±0.79 | 0.237 | 0.871 | / |
|  | 1-4 | 264 | 4.54±0.77 |  |  |  |
|  | 5-9 | 487 | 4.53±0.82 |  |  |  |
|  | ＞9 | 255 | 4.51±0.82 |  |  |  |
| hope | 0 | 257 | 4.43±0.85 | 0.998 | 0.393 | / |
|  | 1-4 | 264 | 4.41±0.82 |  |  |  |
|  | 5-9 | 487 | 4.33±0.88 |  |  |  |
|  | ＞9 | 255 | 4.33±0.88 |  |  |  |
| resilience | 0 | 257 | 4.49±0.87 | 1.442 | 0.229 | / |
|  | 1-4 | 264 | 4.52±0.74 |  |  |  |
|  | 5-9 | 487 | 4.42±0.85 |  |  |  |
|  | ＞9 | 255 | 4.39±0.85 |  |  |  |
| optimism | 0 | 257 | 4.43±0.89 | 1.046 | 0.371 | / |
|  | 1-4 | 264 | 4.41±0.90 |  |  |  |
|  | 5-9 | 487 | 4.38±0.91 |  |  |  |
|  | ＞9 | 255 | 4.30±0.94 |  |  |  |
| Nurse Perceived Organizational Support Scale | 0 | 257 | 3.30±0.73 | 4.683 | 0.003 | 3＞1；3＞2；4＞1；4＞2 |
|  | 1-4 | 264 | 3.24±0.73 |  |  |  |
|  | 5-9 | 487 | 3.42±0.80 |  |  |  |
|  | ＞9 | 255 | 3.44±0.77 |  |  |  |
| emotional support | 0 | 257 | 3.25±0.78 | 4.125 | 0.006 | 3＞2；4＞2 |
|  | 1-4 | 264 | 3.16±0.80 |  |  |  |
|  | 5-9 | 487 | 3.35±0.85 |  |  |  |
|  | ＞9 | 255 | 3.38±0.85 |  |  |  |
| instrumental support | 0 | 257 | 3.47±0.83 | 4.476 | 0.004 | 3＞1；3＞2；4＞1；4＞2 |
|  | 1-4 | 264 | 3.48±0.77 |  |  |  |
|  | 5-9 | 487 | 3.65±0.84 |  |  |  |
|  | ＞9 | 255 | 3.63±0.79 |  |  |  |
| Note：1 means 0, 2 means 1-4, 3 means 5-9, 4 means ＞9. | | | | | | |

## The difference test of the variables in different monthly income (N=1263)

| Scale and its dimensions | group | n | score _mean_（$\bar{x}\pm s$） | *F* value | *P* value | multiple comparisons |
| --- | --- | --- | --- | --- | --- | --- |
| Chinese Nurse Job Stressors Scale | ＜5000 yuan | 405 | 2.59±0.49 | 0.451 | 0.637 | / |
|  | 5000-7999 yuan | 652 | 2.57±0.59 |  |  |  |
|  | ≥8000 yuan | 206 | 2.61±0.57 |  |  |  |
| nursing profession and career issues | ＜5000 yuan | 405 | 2.76±0.64 | 0.420 | 0.657 | // |
|  | 5000-7999 yuan | 652 | 2.73±0.7 |  |  |  |
|  | ≥8000 yuan | 206 | 2.76±0.68 |  |  |  |
| workload and time pressure | ＜5000 yuan | 405 | 2.93±0.63 | 2.551 | 0.078 | / |
|  | 5000-7999 yuan | 652 | 2.86±0.74 |  |  |  |
|  | ≥8000 yuan | 206 | 2.97±0.7 |  |  |  |
| resource and environmental problems | ＜5000 yuan | 405 | 2.42±0.71 | 0.922 | 0.398 | / |
|  | 5000-7999 yuan | 652 | 2.37±0.73 |  |  |  |
|  | ≥8000 yuan | 206 | 2.44±0.76 |  |  |  |
| patient care and interaction | ＜5000 yuan | 405 | 2.51±0.57 | 0.272 | 0.762 |  |
|  | 5000-7999 yuan | 652 | 2.51±0.67 |  |  |  |
|  | ≥8000 yuan | 206 | 2.55±0.66 |  |  |  |
| interpersonal relationships and manage ment issues | ＜5000 yuan | 405 | 2.44±0.66 | 0.072 | 0.930 | / |
|  | 5000-7999 yuan | 652 | 2.42±0.7 |  |  |  |
|  | ≥8000 yuan | 206 | 2.42±0.65 |  |  |  |
| Decisional Fatigue Scale | ＜5000 yuan | 405 | 2.29±0.65 | 0.527 | 0.591 | / |
|  | 5000-7999 yuan | 652 | 2.27±0.66 |  |  |  |
|  | ≥8000 yuan | 206 | 2.32±0.68 |  |  |  |
| Nurse Psychological Capital Questionnaire | ＜5000 yuan | 405 | 4.45±0.68 | 0.290 | 0.748 | / |
|  | 5000-7999 yuan | 652 | 4.44±0.72 |  |  |  |
|  | ≥8000 yuan | 206 | 4.41±0.68 |  |  |  |
| self-efficacy | ＜5000 yuan | 405 | 4.57±0.8 | 0.592 | 0.553 | / |
|  | 5000-7999 yuan | 652 | 4.53±0.81 |  |  |  |
|  | ≥8000 yuan | 206 | 4.51±0.79 |  |  |  |
| hope | ＜5000 yuan | 405 | 4.38±0.84 | 0.036 | 0.965 | / |
|  | 5000-7999 yuan | 652 | 4.37±0.87 |  |  |  |
|  | ≥8000 yuan | 206 | 4.36±0.87 |  |  |  |
| resilience | ＜5000 yuan | 405 | 4.45±0.81 | 0.637 | 0.529 | / |
|  | 5000-7999 yuan | 652 | 4.46±0.86 |  |  |  |
|  | ≥8000 yuan | 206 | 4.39±0.81 |  |  |  |
| optimism | ＜5000 yuan | 405 | 4.37±0.92 | 0.486 | 0.615 | / |
|  | 5000-7999 yuan | 652 | 4.4±0.93 |  |  |  |
|  | ≥8000 yuan | 206 | 4.34±0.84 |  |  |  |
| Nurse Perceived Organizational Support Scale | ＜5000 yuan | 405 | 3.18±0.79 | 28.902 | 0.000 | 2＞1；3＞1；3＞2 |
|  | 5000-7999 yuan | 652 | 3.38±0.73 |  |  |  |
|  | ≥8000 yuan | 206 | 3.66±0.75 |  |  |  |
| emotional support | ＜5000 yuan | 405 | 3.1±0.85 | 29.395 | 0.000 | 2＞1；3＞1；3＞2 |
|  | 5000-7999 yuan | 652 | 3.32±0.79 |  |  |  |
|  | ≥8000 yuan | 206 | 3.63±0.8 |  |  |  |
| instrumental support | ＜5000 yuan | 405 | 3.43±0.81 | 12.888 | 0.000 | 2＞1；3＞1；3＞2 |
|  | 5000-7999 yuan | 652 | 3.61±0.81 |  |  |  |
|  | ≥8000 yuan | 206 | 3.76±0.81 |  |  |  |
| Note：1 means ＜5000 yuan, 2 means 5000-7999 yuan, 3 means ≥8000 yuan. | | | | | | |

## The difference test of the variables in different marital status(N=1263)

| Scale and its dimensions | group | n | score _mean_（$\bar{x}\pm s$） | *F* value | *P* value | multiple comparisons |
| --- | --- | --- | --- | --- | --- | --- |
| Chinese Nurse Job Stressors Scale | unmarried | 312 | 2.55±0.52 | 0.497 | 0.685 | / |
|  | married | 919 | 2.6±0.57 |  |  |  |
|  | divorced | 31 | 2.56±0.57 |  |  |  |
|  | widowed | 1 | 2.43±0.00 |  |  |  |
| nursing profession and career issues | unmarried | 312 | 2.74±0.63 | 0.151 | 0.929 | / |
|  | married | 919 | 2.75±0.69 |  |  |  |
|  | divorced | 31 | 2.66±0.7 |  |  |  |
|  | widowed | 1 | 2.71±0.00 |  |  |  |
| workload and time pressure | unmarried | 312 | 2.87±0.66 | 0.572 | 0.633 | / |
|  | married | 919 | 2.91±0.71 |  |  |  |
|  | divorced | 31 | 2.99±0.66 |  |  |  |
|  | widowed | 1 | 2.4±0.00 |  |  |  |
| resource and environmental problems | unmarried | 312 | 2.39±0.68 | 0.851 | 0.466 | / |
|  | married | 919 | 2.41±0.74 |  |  |  |
|  | divorced | 31 | 2.33±0.87 |  |  |  |
|  | widowed | 1 | 1.33±0.00 |  |  |  |
| patient care and interaction | unmarried | 312 | 2.51±0.61 | 0.082 | 0.97 | / |
|  | married | 919 | 2.52±0.65 |  |  |  |
|  | divorced | 31 | 2.49±0.69 |  |  |  |
|  | widowed | 1 | 2.55±0.00 |  |  |  |
| interpersonal relationships and manage ment issues | unmarried | 312 | 2.34±0.67 | 2.227 | 0.083 | / |
|  | married | 919 | 2.46±0.68 |  |  |  |
|  | divorced | 31 | 2.4±0.58 |  |  |  |
|  | widowed | 1 | 2.44±0.00 |  |  |  |
| Decisional Fatigue Scale | unmarried | 312 | 2.28±0.63 | 0.636 | 0.592 | / |
|  | married | 919 | 2.29±0.67 |  |  |  |
|  | divorced | 31 | 2.24±0.61 |  |  |  |
|  | widowed | 1 | 1.44±0.00 |  |  |  |
| Nurse Psychological Capital Questionnaire | unmarried | 312 | 4.44±0.68 | 1.172 | 0.319 | / |
|  | married | 919 | 4.44±0.71 |  |  |  |
|  | divorced | 31 | 4.27±0.63 |  |  |  |
|  | widowed | 1 | 5.35±0.00 |  |  |  |
| self-efficacy | unmarried | 312 | 4.52±0.83 | 1.035 | 0.376 | / |
|  | married | 919 | 4.55±0.8 |  |  |  |
|  | divorced | 31 | 4.38±0.58 |  |  |  |
|  | widowed | 1 | 5.50±0.00 |  |  |  |
| hope | unmarried | 312 | 4.39±0.8 | 1.208 | 0.306 | / |
|  | married | 919 | 4.36±0.89 |  |  |  |
|  | divorced | 31 | 4.20±0.76 |  |  |  |
|  | widowed | 1 | 5.67±0.00 |  |  |  |
| resilience | unmarried | 312 | 4.46±0.79 | 0.564 | 0.639 | / |
|  | married | 919 | 4.45±0.85 |  |  |  |
|  | divorced | 31 | 4.32±0.91 |  |  |  |
|  | widowed | 1 | 5.20±0.00 |  |  |  |
| optimism | unmarried | 312 | 4.38±0.9 | 0.939 | 0.421 | / |
|  | married | 919 | 4.39±0.91 |  |  |  |
|  | divorced | 31 | 4.12±0.94 |  |  |  |
|  | widowed | 1 | 4.67±0.00 |  |  |  |
| Nurse Perceived Organizational Support Scale | unmarried | 312 | 3.15±0.70 | 11.64 | 0.000 | / |
|  | married | 919 | 3.44±0.78 |  |  |  |
|  | divorced | 31 | 3.29±0.67 |  |  |  |
|  | widowed | 1 | 2.92±0.00 |  |  |  |
| emotional support | unmarried | 312 | 3.06±0.77 | 11.918 | 0.000 | / |
|  | married | 919 | 3.38±0.84 |  |  |  |
|  | divorced | 31 | 3.20±0.69 |  |  |  |
|  | widowed | 1 | 2.90±0.00 |  |  |  |
| instrumental support | unmarried | 312 | 3.42±0.76 | 5.014 | 0.002 | / |
|  | married | 919 | 3.63±0.83 |  |  |  |
|  | divorced | 31 | 3.58±0.83 |  |  |  |
|  | widowed | 1 | 3.00±0.00 |  |  |  |
| Note：1 means unmarried, 2 means married, 3 means divorced, 4 means widowed. | | | | | | |

## The difference test of the variables in different fertility status (N=1263)

| Scale and its dimensions | group | n | score _mean_（$\bar{x}\pm s$） | *F* value | *P* value | multiple comparisons |
| --- | --- | --- | --- | --- | --- | --- |
| Chinese Nurse Job Stressors Scale | none | 398 | 2.57±0.54 | 0.363 | 0.78 | / |
|  | one child | 568 | 2.59±0.56 |  |  |  |
|  | two children | 291 | 2.59±0.58 |  |  |  |
|  | three or more children | 6 | 2.42±0.49 |  |  |  |
| nursing profession and career issues | none | 398 | 2.75±0.65 | 0.259 | 0.855 | / |
|  | one child | 568 | 2.74±0.69 |  |  |  |
|  | two children | 291 | 2.76±0.68 |  |  |  |
|  | three or more children | 6 | 2.52±0.59 |  |  |  |
| workload and time pressure | none | 398 | 2.88±0.69 | 0.422 | 0.737 | / |
|  | one child | 568 | 2.92±0.7 |  |  |  |
|  | two children | 291 | 2.88±0.71 |  |  |  |
|  | three or more children | 6 | 3.07±0.59 |  |  |  |
| resource and environmental problems | none | 398 | 2.4±0.7 | 1.304 | 0.272 | / |
|  | one child | 568 | 2.38±0.74 |  |  |  |
|  | two children | 291 | 2.45±0.75 |  |  |  |
|  | three or more children | 6 | 1.94±0.49 |  |  |  |
| patient care and interaction | none | 398 | 2.51±0.63 | 0.674 | 0.568 | / |
|  | one child | 568 | 2.53±0.63 |  |  |  |
|  | two children | 291 | 2.51±0.67 |  |  |  |
|  | three or more children | 6 | 2.2±0.58 |  |  |  |
| interpersonal relationships and manage ment issues | none | 398 | 2.38±0.67 | 0.957 | 0.412 | / |
|  | one child | 568 | 2.44±0.69 |  |  |  |
|  | two children | 291 | 2.46±0.67 |  |  |  |
|  | three or more children | 6 | 2.41±0.54 |  |  |  |
| Decisional Fatigue Scale | none | 398 | 2.28±0.64 | 1.219 | 0.302 | / |
|  | one child | 568 | 2.27±0.65 |  |  |  |
|  | two children | 291 | 2.33±0.68 |  |  |  |
|  | three or more children | 6 | 1.93±0.75 |  |  |  |
| Nurse Psychological Capital Questionnaire | none | 398 | 4.47±0.69 | 0.311 | 0.817 | / |
|  | one child | 568 | 4.43±0.72 |  |  |  |
|  | two children | 291 | 4.43±0.7 |  |  |  |
|  | three or more children | 6 | 4.28±0.71 |  |  |  |
| self-efficacy | none | 398 | 4.55±0.83 | 0.152 | 0.928 | / |
|  | one child | 568 | 4.54±0.79 |  |  |  |
|  | two children | 291 | 4.51±0.8 |  |  |  |
|  | three or more children | 6 | 4.58±0.71 |  |  |  |
| hope | none | 398 | 4.4±0.8 | 0.316 | 0.814 | / |
|  | one child | 568 | 4.35±0.9 |  |  |  |
|  | two children | 291 | 4.37±0.87 |  |  |  |
|  | three or more children | 6 | 4.28±0.79 |  |  |  |
| resilience | none | 398 | 4.49±0.81 | 0.83 | 0.477 | / |
|  | one child | 568 | 4.43±0.85 |  |  |  |
|  | two children | 291 | 4.42±0.82 |  |  |  |
|  | three or more children | 6 | 4.1±1.22 |  |  |  |
| optimism | none | 398 | 4.38±0.91 | 0.365 | 0.778 | / |
|  | one child | 568 | 4.38±0.9 |  |  |  |
|  | two children | 291 | 4.39±0.93 |  |  |  |
|  | three or more children | 6 | 4±0.63 |  |  |  |
| Nurse Perceived Organizational Support Scale | none | 398 | 3.2±0.73 | 10.228 | 0.000 | 2＞1；3＞1 |
|  | one child | 568 | 3.41±0.79 |  |  |  |
|  | two children | 291 | 3.49±0.76 |  |  |  |
|  | three or more children | 6 | 3.06±0.55 |  |  |  |
| emotional support | none | 398 | 3.12±0.79 | 10.476 | 0.000 | 2＞1；3＞1 |
|  | one child | 568 | 3.35±0.84 |  |  |  |
|  | two children | 291 | 3.44±0.81 |  |  |  |
|  | three or more children | 6 | 2.87±0.67 |  |  |  |
| instrumental support | none | 398 | 3.45±0.78 | 4.871 | 0.002 | 2＞1；3＞1 |
|  | one child | 568 | 3.62±0.84 |  |  |  |
|  | two children | 291 | 3.65±0.82 |  |  |  |
|  | three or more children | 6 | 3.72±0.39 |  |  |  |
| Note：1 means none, 2 means one child, 3 means two children, 4 means three or more children. | | | | | | |
